# Supplementary material for: Distinct immune cell infiltration patterns in pancreatic ductal adenocarcinoma (PDAC) exhibit divergent immune cell selection and immunosuppressive mechanisms
Source: Nat Commun. 2025 Feb 6;16:1397. doi: 10.1038/s41467-024-55424-2 (PMC11802853; doi:10.1038/s41467-024-55424-2)
Supplement: Supplementary file 1 — Supplementary Information [file 41467_2024_55424_MOESM1_ESM.pdf]

Title: Distinct immune cell infiltration patterns in pancreatic ductal adenocarcinoma (PDAC) exhibit divergent immune cell selection and immunosuppressive mechanisms

Shivan Sivakumar*1,2,3, Ashwin Jainarayanan*2,4, Edward Arbe-Barnes*5, Piyush Kumar Sharma+1, Maire Ni Leathlobhair+6,7, Sakina Amin+8, David J Reiss+9, Lara Heij10,11, Samarth Hegde12, Assaf Magen12, Felicia Tucci8,13,14, Bo Sun15, Shihong Wu8,13, Nithishwer Mouroug Anand8, Hubert Slawinski14, Santiago Revale14, Isar Nassiri14, Jonathon Webber2, Gerard D. Hoeltzel8, Adam Frampton16,17,18,19, Georg Wiltberger20, Ulf Neumann21, Philip Charlton1, Laura Spiers1, Tim Elliott22, Maria Wang9, Suzana Couto23, Thomas Lila9, Pallavur V. Sivakumar9, Alexander V. Ratushny9, Mark Middleton1, Dimitra Peppa24,25, Benjamin Fairfax1, Miriam Merad12, Michael L. Dustin2,26§, Enas Abu-Shah2,27§, Rachael Bashford-Rogers8,13,14§

Supplemental Figures.

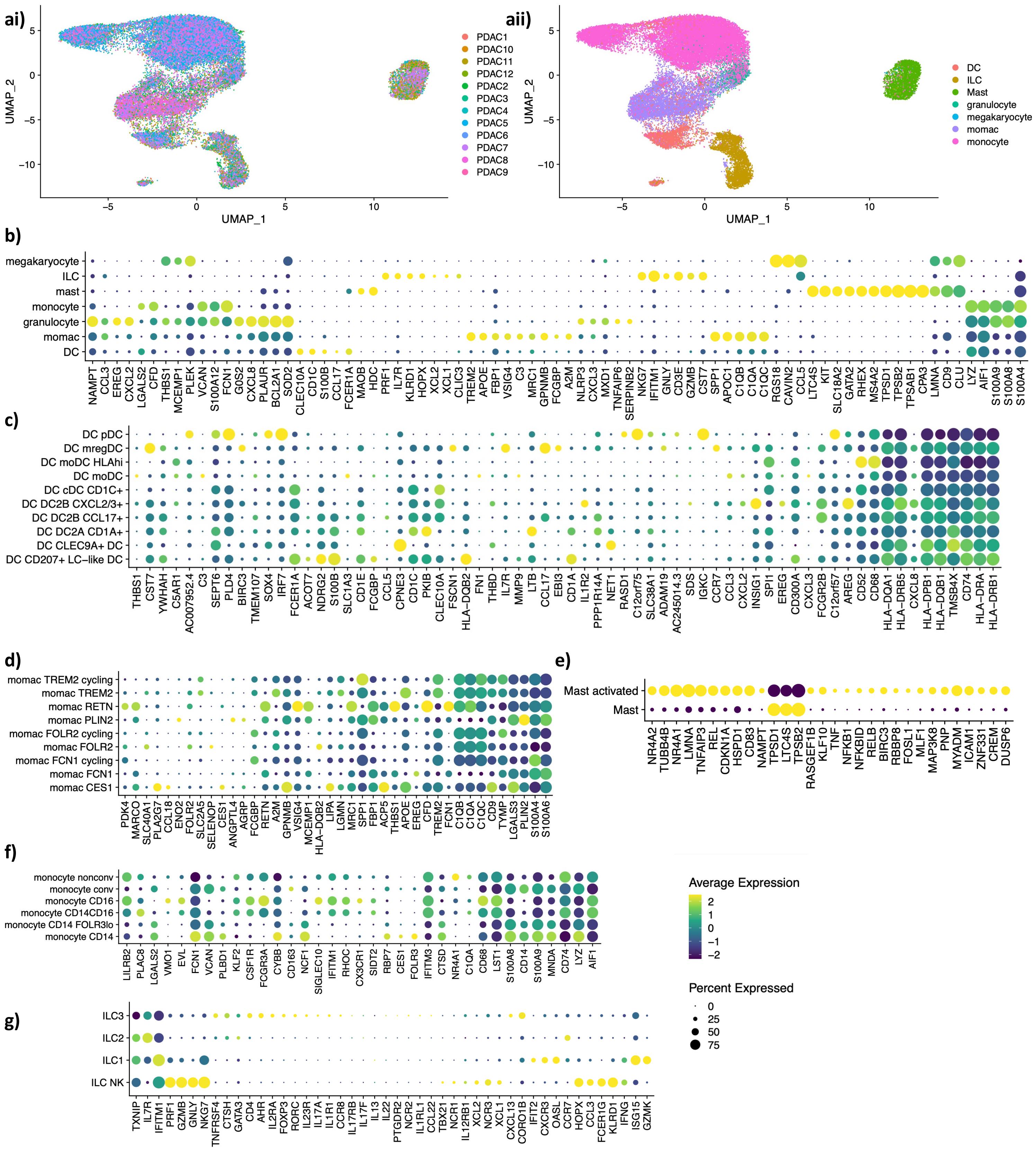

Supplementary Figure 1

Supplemental Figure 1. Gene signatures for the myeloid cell annotations
a) Tumour myeloid UMAP coloured by (i) patient, and (ii) broad myeloid cell type
Gene expression signatures of (b) broad myeloid cell types, (c) DC, (d) monocyte-derived macrophages (momac), (e) mast cell, (f) monocyte, and g) ILC subpopulations.
The suffix “b” indicates blood-derived populations.

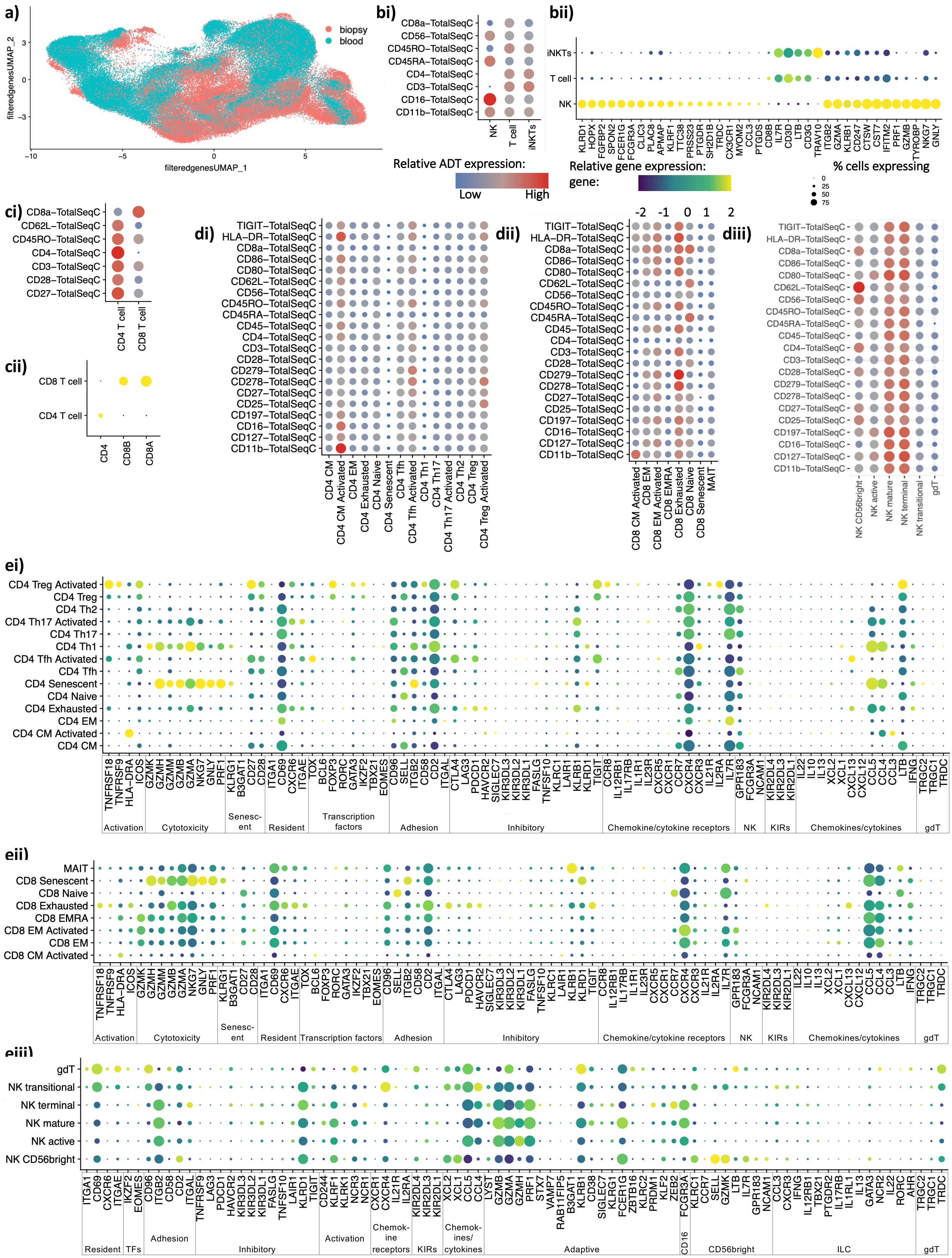

Supplementary Figure 2

Supplemental Figure 2. Gene signatures for the T/NK cell annotations
a) UMAP coloured by sample source.
b) (i) CITE-seq and (ii) gene expression signatures of NK versus T cells.
c) (i) CITE-seq and (ii) gene expression signatures of CD4 versus CD8 T cells.
d) CITE-seq signatures of (i) CD4 T cell, (ii) CD8 T cell and (iii) NK cell populations. CITE-seq values are scaled by maximum value per cell type group.
e) Gene expression signatures of (i) CD4 T cell, (ii) CD8 T cell and (iii) NK cell populations.

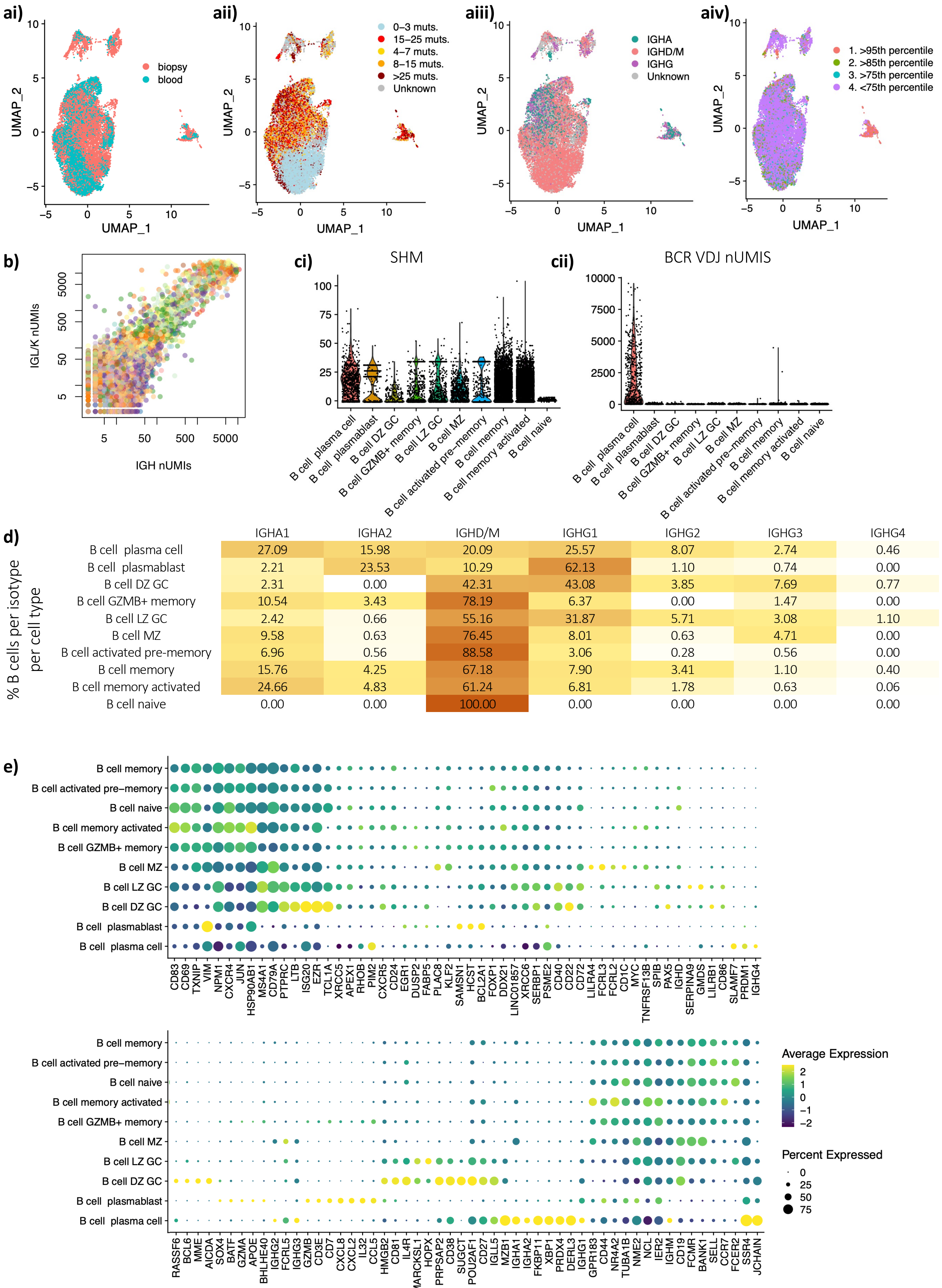

Supplementary Figure 3

Supplemental Figure 3. Gene and VDJ signatures for the B cell annotations.
a) UMAP plots of B cells coloured by (i) source, (ii) somatic hypermutation level, (iii) isotype and (iv) VDJ expression level.
b) Correlation of IGH and IGK/L UMI counts per cell, coloured by PDAC sample type.
c) The per cell subpopulation (i) somatic hypermutation levels and ii) VDJ expression level.
d) The isotype usage percentages across cell types within each cell population.
e) Gene expression profiles of B cell subpopulations of the top differentially expressed genes.

Clone size distributions across cell types (intra-tumoural samples)

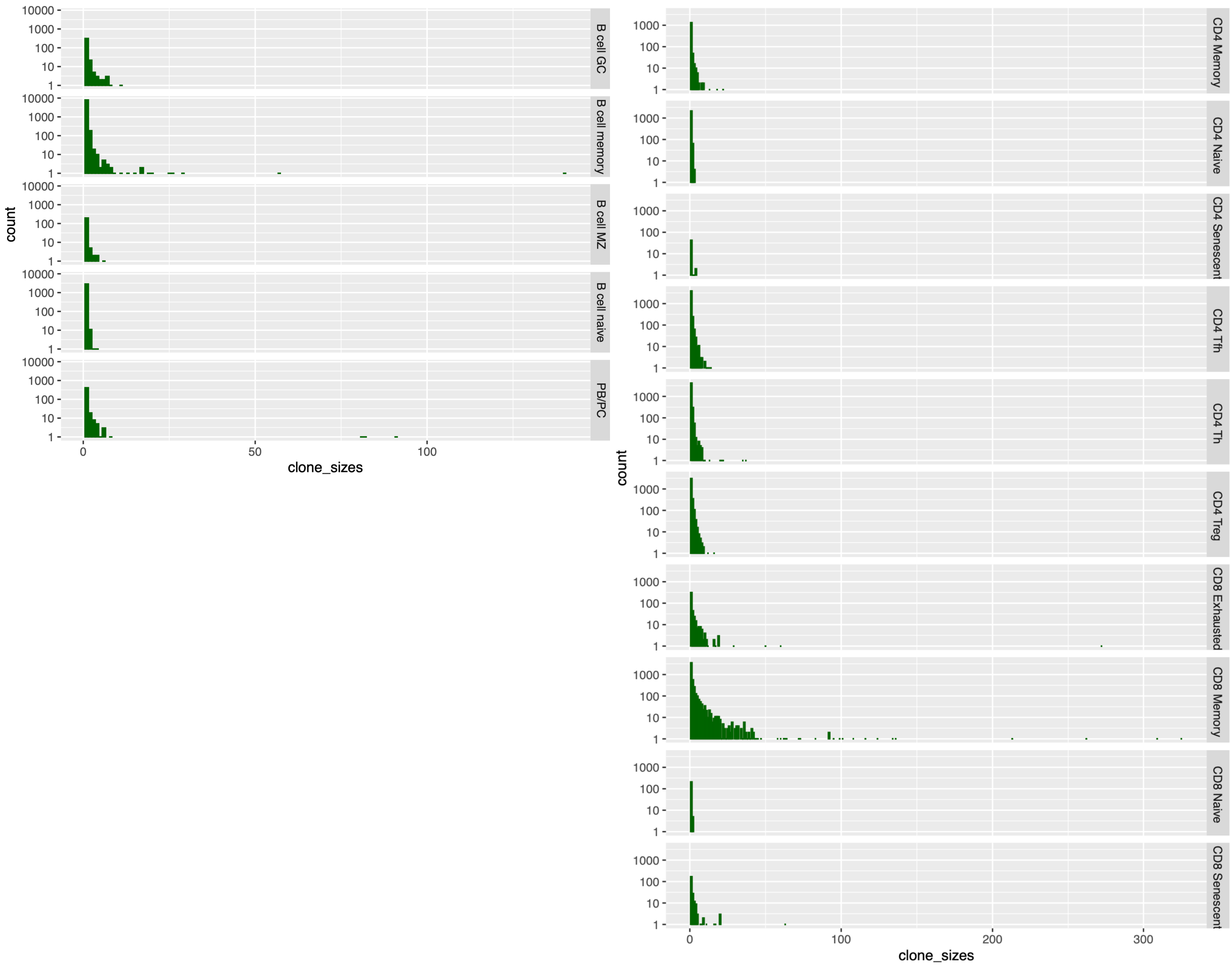

Supplementary Figure 4

Supplemental Figure 4. Clone size distributions across cell types for (left) B cell and (right) T cell populations, considering only intra-tumoural immune cells.

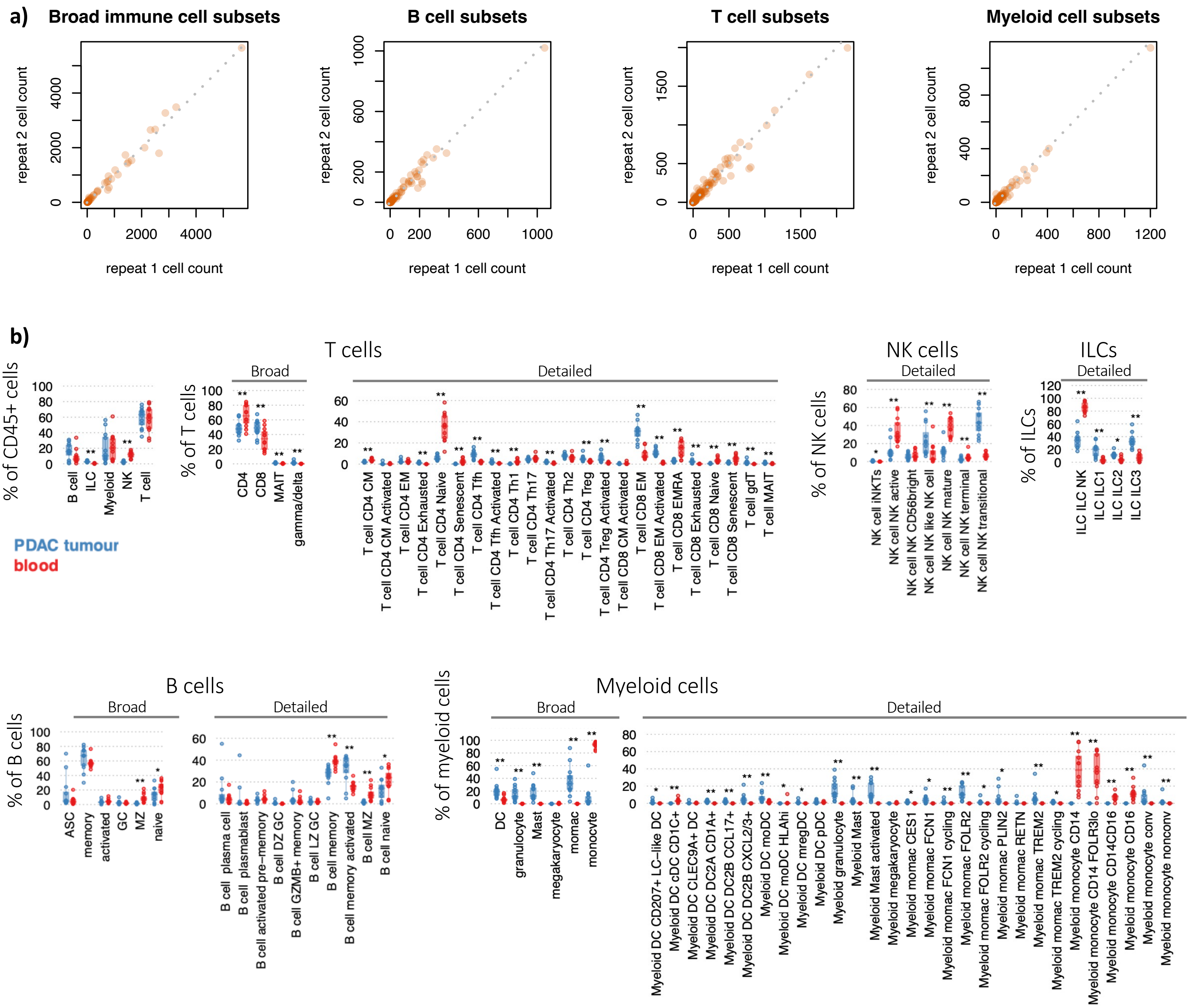

Supplementary Figure 5

Supplemental Figure 5

a) Correlation of cell types between technical repeats of biopsy samples for the broad immune cell subsets, B cell subsets, T cell subsets and myeloid cell subsets.

b) Boxplots of differences between biopsy and blood immune cell proportions. Each dot represents a patient sample.

a) **SVMCellTransfer advantages over established methods:**

Annotations of the reference can be performed using cell type expertise and/or integration of multi-modal single cell data, using datasets that reflect well the query dataset

The unbiased cell type annotation ensures that cell annotation is not based on probability of higher number of a cell type but based on gene expression. This can also speed up the subsequent steps when using a large reference.

Allows the user to define how the reference and query datasets are integrated prior to label transfer

Scalable, fast, does not rely on user-defined markers, considers non-linear relationships and does not rely on a web-server interface.

High-confidence user-defined annotated reference dataset (PancrImmune)

Query unannotated dataset

(Optional) **Subsample** each cell type from reference dataset

Reference and query dataset overlay (integration, batch correction, dimensionality reduction)

Run SVMCellTransfer using an SVM to label unknown cells using the dimensionality reduction distribution with the reference dataset:
Step 1: build model of cell type distributions using the labelled data
Step 2: use this model to predict unknown labels, providing the most likely cell type and the posterior probability for each cell.

b)

T/NK cells from **Peng dataset** (subsampled random 6,925 cells) and **Steele dataset** (subsampled random 4,703 cells)

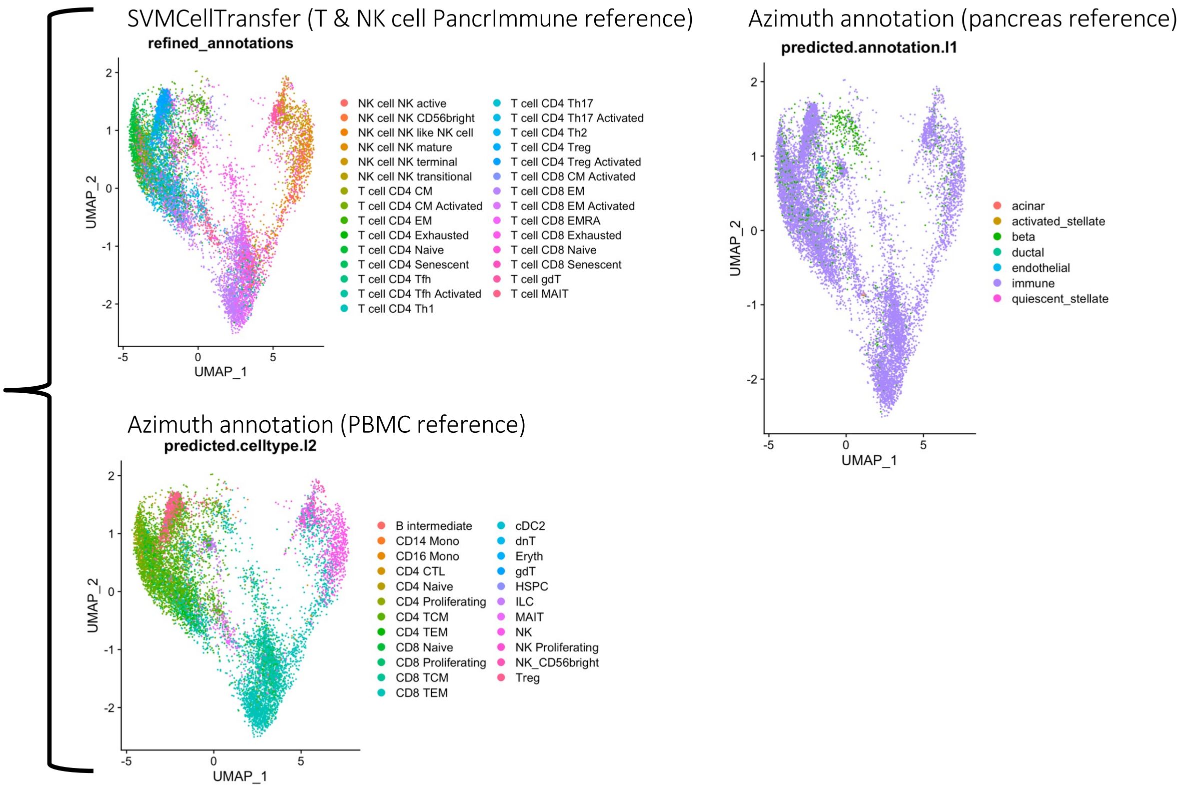

Supplementary Figure 6

Supplemental Figure 6. a) Schematic of SVMCellTransfer alongside the advantages of this method over established methods (left).
b) Comparison of reference annotation of T and NK cells using SVMCellTransfer (using T and NK cell PancrImmune reference), and Azimuth annotation (using either pancreas or PBMC references). High-confidence T and NK cells were subsampled from the Peng and Steele datasets (confirmed by manual checking of key T and NK genes), and applied both the SVMCellTransfer (using T and NK cell PancrImmune reference), and Azimuth annotation (using either pancreas or PBMC references). UMAP plots show the distribution of predicted cell types by each method.

Integrated PDAC150K, Peng, *et al.* and Steele, *et al.* datasets

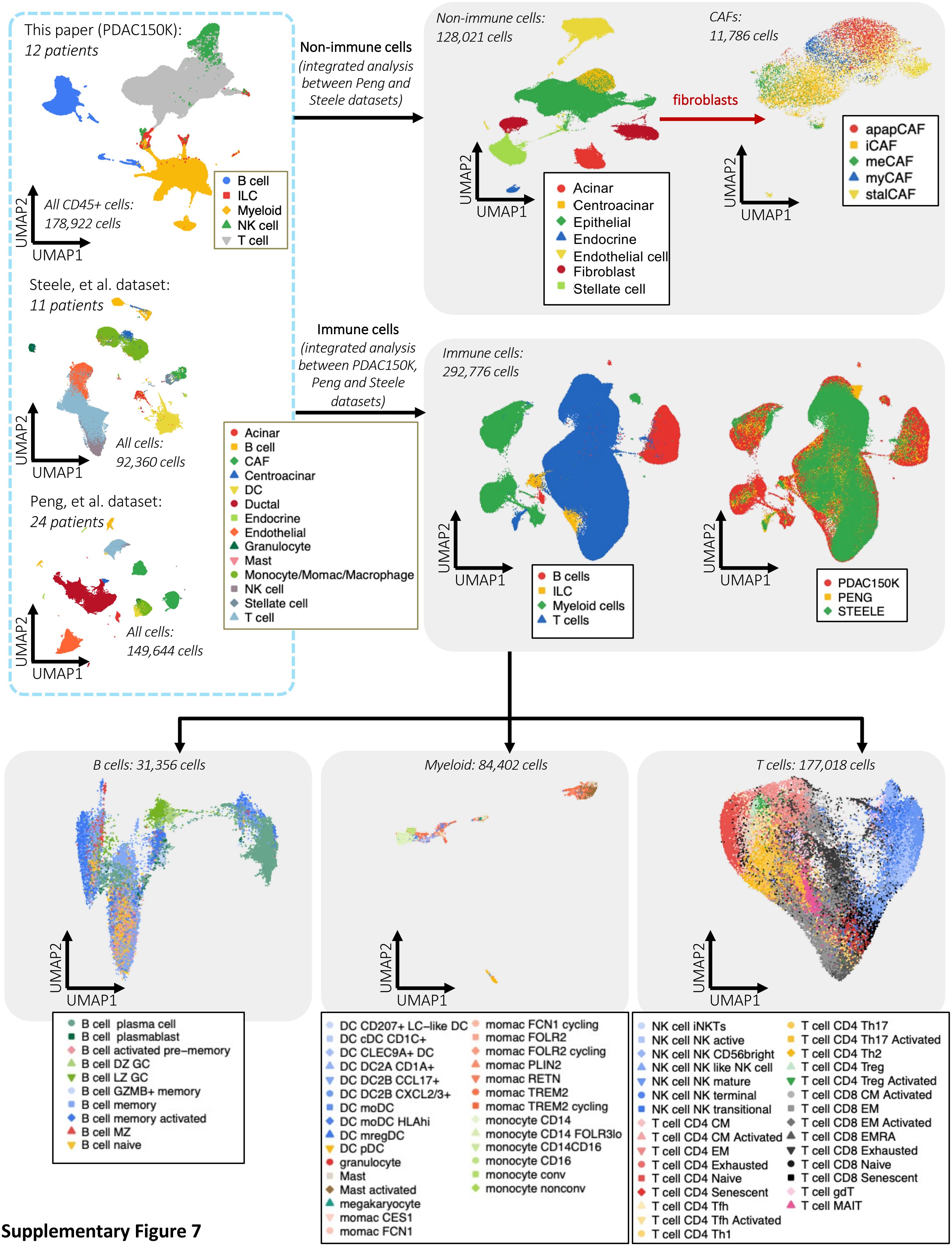

Supplementary Figure 7

Supplemental Figure 7. UMAP distributions of the integrated PDAC150K, Peng, et al. and Steele, et al. datasets.

ai) Immune cells *(integrated analysis between PDAC150K, Peng and Steele datasets)*

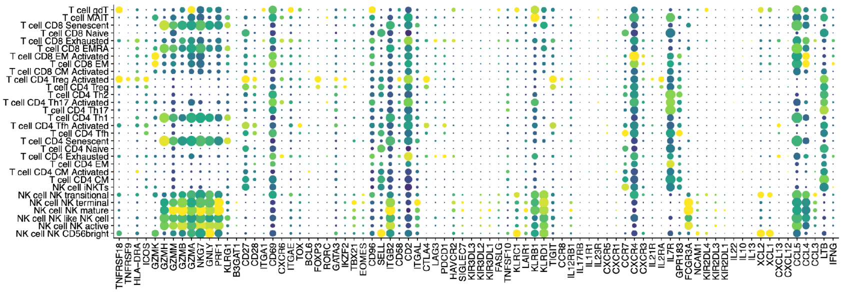

ii)

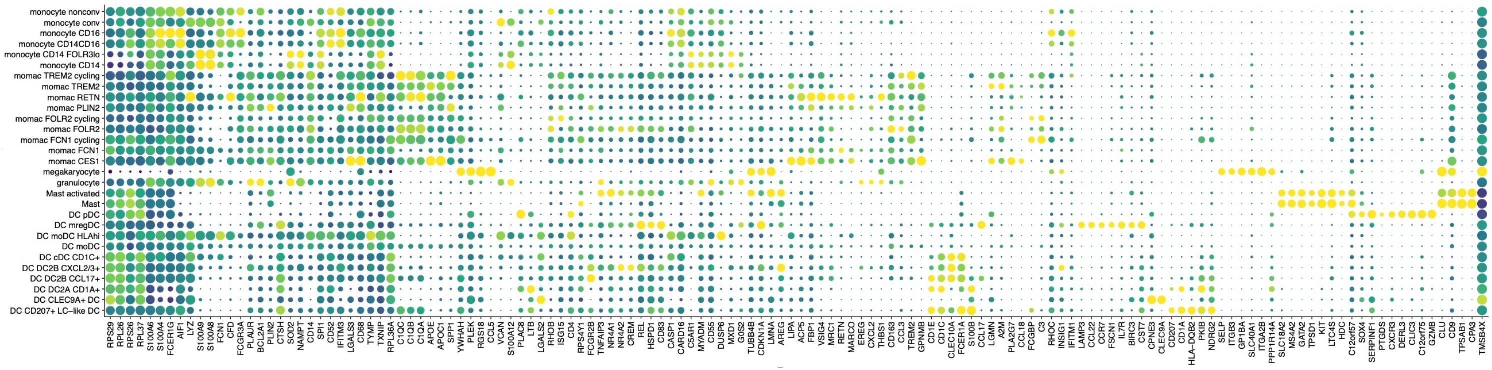

iii)

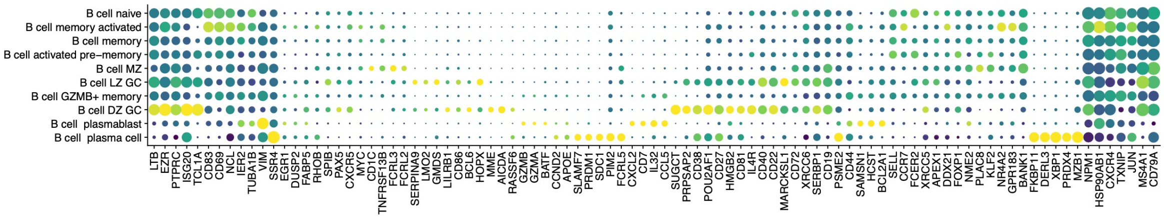

bi) Non-immune cells *(integrated analysis between Peng and Steele datasets)*

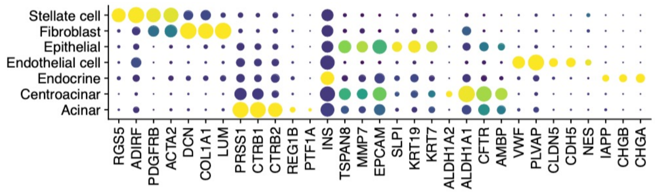

ii)

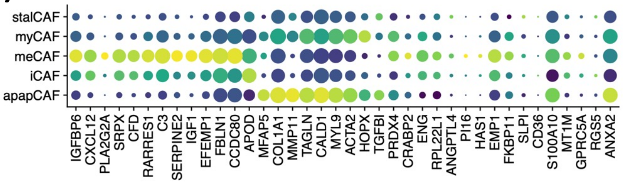

Supplementary Figure 8

Supplemental Figure 8. Gene expression signatures of (ai) T and NK cell types, (ii) DC, (d) myeloid cell types, (iii) B cell subtypes, (bi) non-immune cell types, and (ii) CAF cell types of the integrated PDAC150K, Peng, et al. and Steele, et al. datasets.

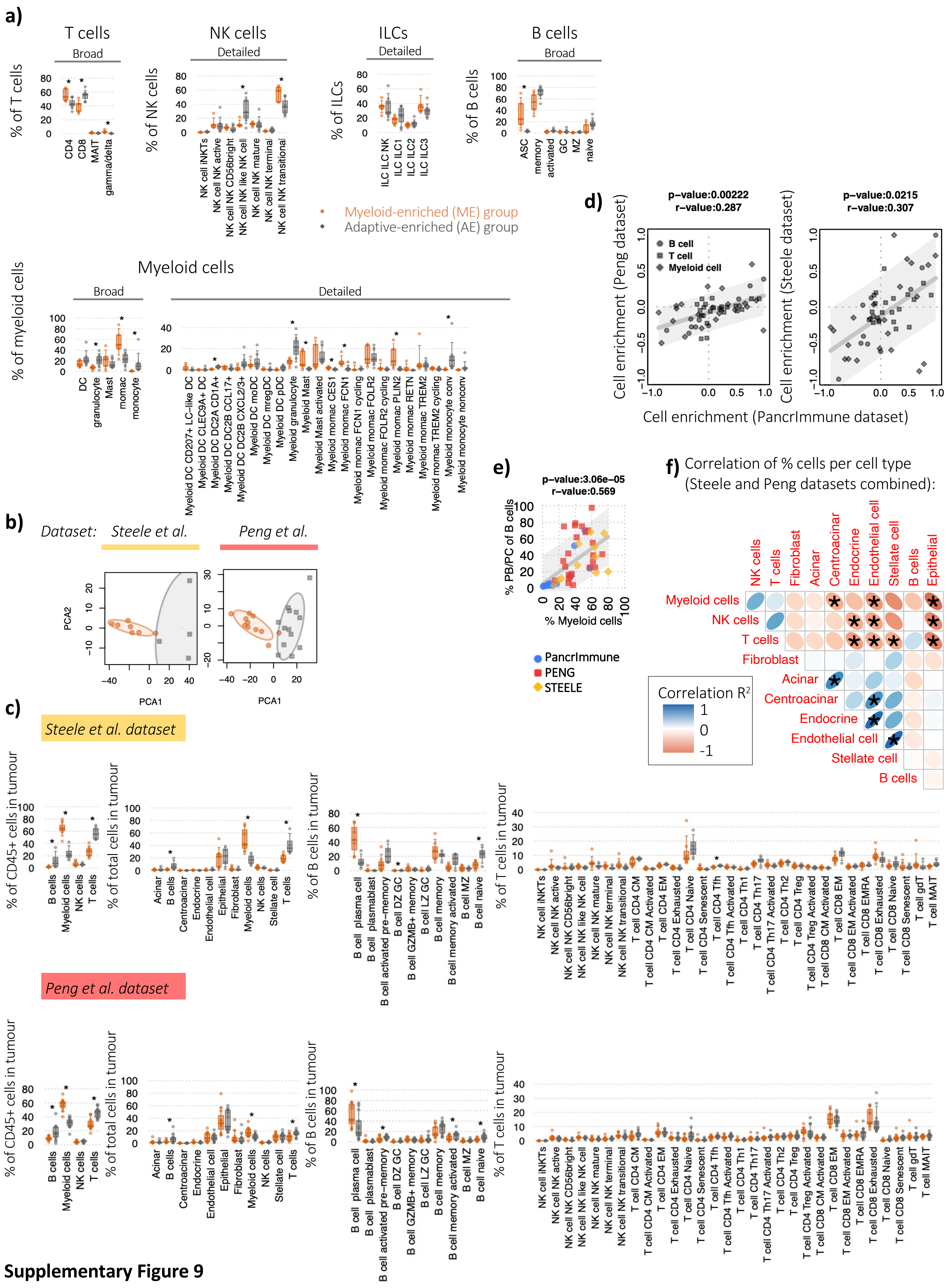

Supplementary Figure 9

Supplementary Figure 9: Increased intra-tumoural lymphocyte infiltration is associated with distinct immune cellular compositions.

a) Tumour immune cell subset proportions between ME and AE patient groups within cellular subsets as a proportion within the PancrImmune dataset. Orange represents ME patients and grey represents AE patients.

b) Principal component analysis (PCA) based on PDAC CD45+ immune cell infiltration proportions for the Steele and Peng datasets, coloured by patient group.

c) Cell subset proportions between ME and AE patient groups within cellular subsets as a proportion for the Steele and Peng datasets. Orange represents ME patients and grey represents AE patients.

d) Correlation of the cell enrichment between ME and AE patients between the PancrImmune tumour and (left) Peng and (right) Steele datasets. P-values and R^2 values provided above each plot.

e) The correlation of myeloid cells as a proportion of total intra-tumoural immune cells with plasmablasts and plasma cells as a proportion of total B cells, coloured blue, red and yellow for the PancrImmune, Peng and Steele datasets respectively.

f) Correlation of immune and non-immune cell proportions from the Steele and Peng datasets combined as a proportion of total cells. The blue positively sloped ellipses represent positive correlations and negatively sloped ellipses represent negative correlations, and * denotes significant correlations p-values<0.05.

In panels (a) and (c), * denotes p-values<0.05, and tests were performed by two-sided MANOVA. All analyses were performed on intra-tumoural cells. ME patients have an n=5 (PancrImmune), n=11 (Peng) and n=7 (Steele), and AE patients have an n=7 (PancrImmune), n=13 (Peng) and n=4 (Steele).

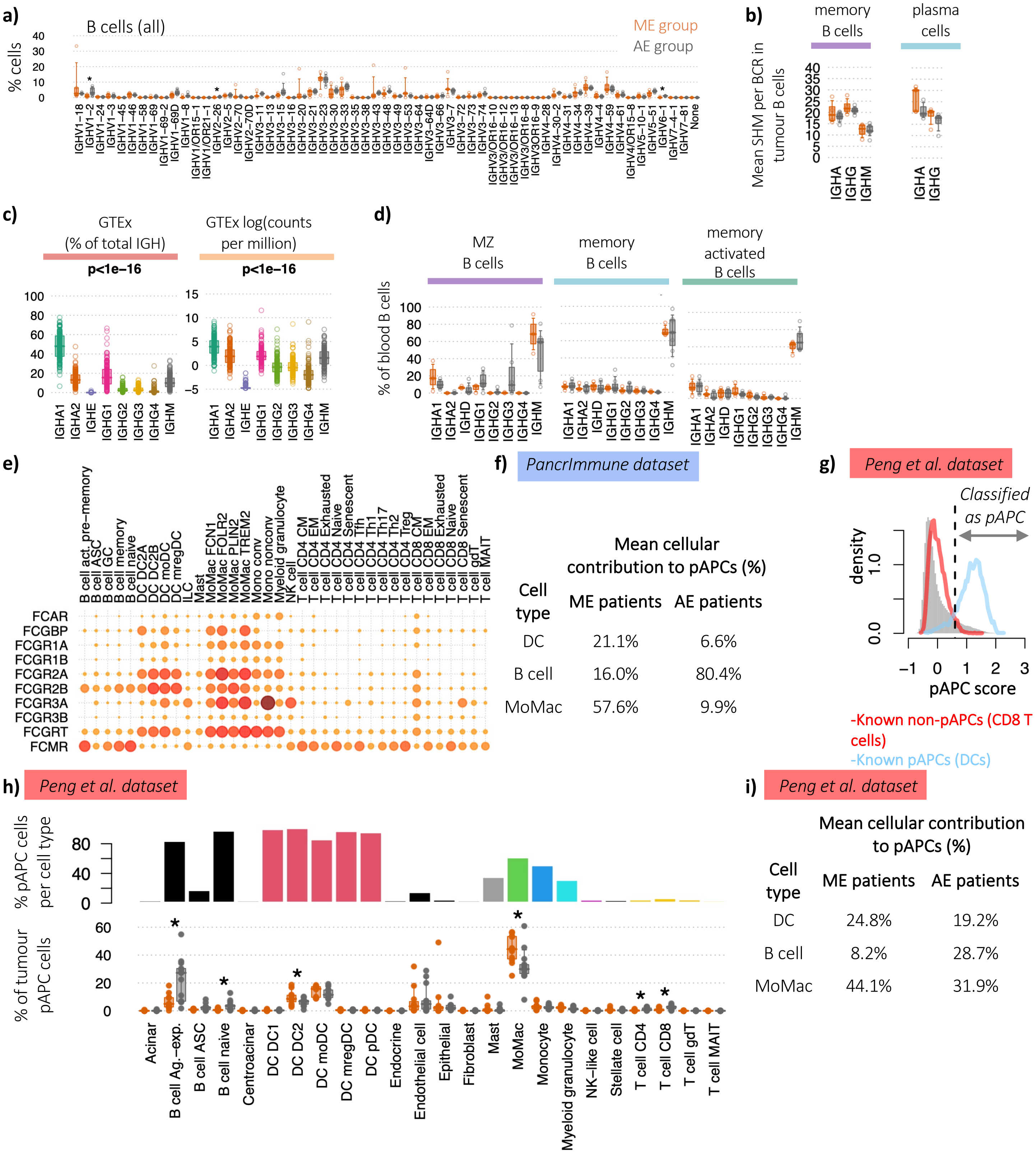

f) PancrImmune dataset

Cell type	Mean cellular contribution to pAPCs (%) ME patients	AE patients
DC	21.1%	6.6%
B cell	16.0%	80.4%
MoMac	57.6%	9.9%

i) Peng et al. dataset

Cell type	Mean cellular contribution to pAPCs (%) ME patients	AE patients
DC	24.8%	19.2%
B cell	8.2%	28.7%
MoMac	44.1%	31.9%

Supplementary Figure 10

Supplementary Figure 10: Increased PDAC lymphocyte infiltration is associated with differences in B cell selection, clonal expansion and class-switch recombination.
a) IGHV proportions between ME and AE patient groups of total tumour B cells within the PancrImmune dataset. Orange represents ME patients and grey represents AE patients.
b) Mean SHM levels between ME and AE patient groups for tumour memory B cells and plasma cells within the PancrImmune dataset.
c) Isotype usages (left) as a proportion of total IGH and (right) counts per million in healthy pancreatic tissue from the GTEx RNA-seq dataset. P-values generated by ANOVA.
d) The proportions of blood B cells within activated, memory and plasma cells expressing each isotype, coloured by patient group using the PancrImmune dataset.
e) The relative levels of the FC receptor gene expression between intra-tumoural cell types, where larger circle size indicates higher expression using the PancrImmune dataset.
f) Table of the mean cellular contribution to pAPCs between ME and AE patient groups using the PancrImmune dataset.
g) Histogram of the professional antigen presentation (pAPC) scores for (grey) all cells, (red) CD8 T cells and (blue) DCs, using the Peng dataset. Dashed line indicates the threshold for classification of pAPCs.
h) (top) Barchart of the percentages of pAPCs comprising each cell type, and (bottom) the proportion of pAPCs comprising each cell type between patient groups, using the Peng dataset.
i) Table of the mean cellular contribution to pAPCs between ME and AE patient groups, using the Peng dataset.
All analyses were performed on intra-tumoural cells. * denotes p-values<0.05, and tests were performed by two-sided MANOVA. ME patients have an n=5 (PancrImmune), n=11 (Peng) and n=7 (Steele), and AE patients have an n=7 (PancrImmune), n=13 (Peng) and n=4 (Steele).

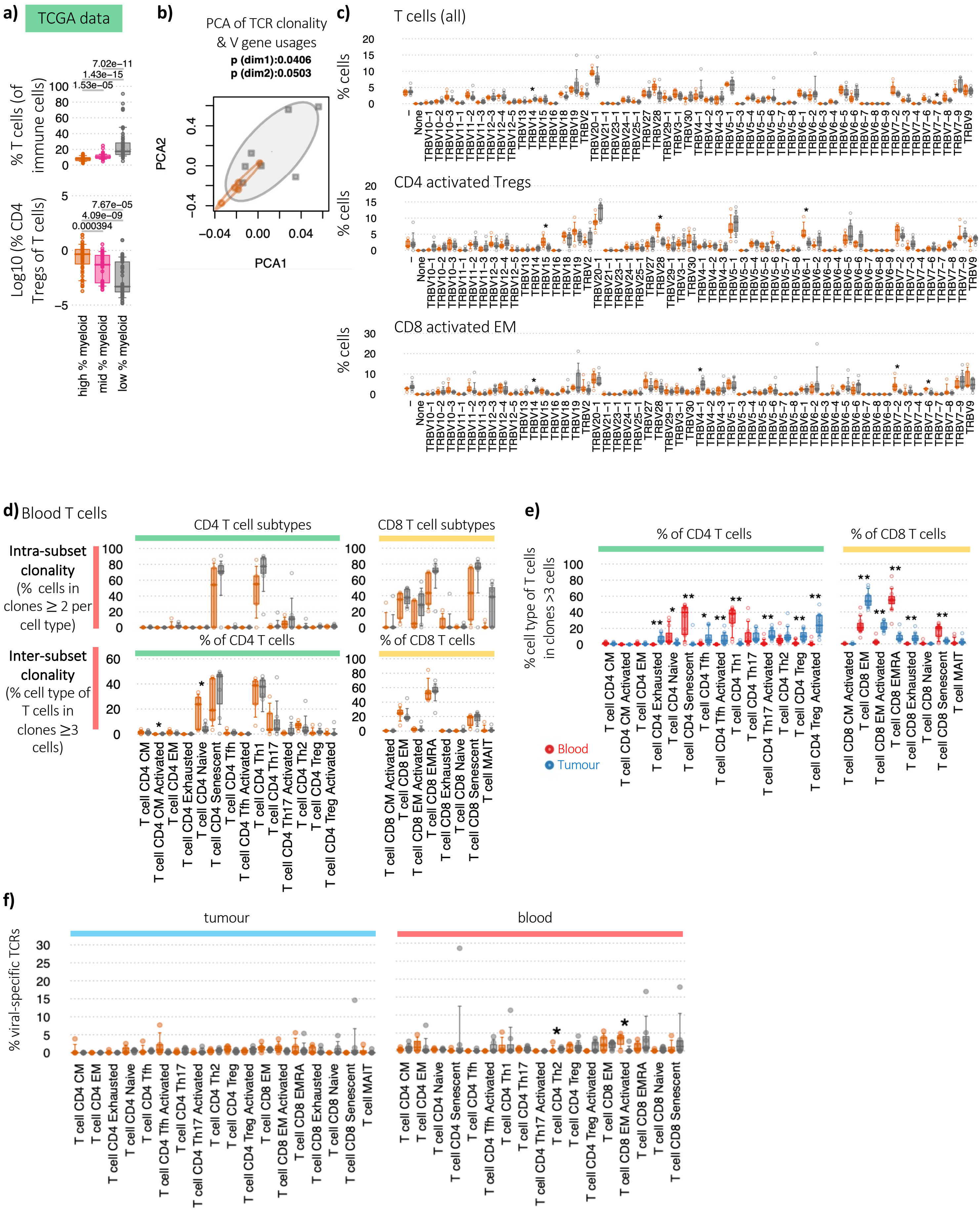

Supplementary Figure 11

Supplementary Figure 11: Increased PDAC lymphocyte infiltration is associated with increased activated Treg clonality

a) Correlation of the Treg proportions (as a proportion of total T cells) with myeloid cell proportions (as a proportion of total immune cells). Cellular deconvolution of the PAAD TCGA dataset (n=156 patients) by BayesPrism using the PancrImmune dataset as a reference. The TCGA patients were split into tertials based on myeloid cell proportions (low % myeloid cells = lowest 33% of patients, mid % myeloid cells = mid 33% of patients, high % myeloid cells = highest 33% of patients). P-values calculated by Wilcoxon test.

b) Principal component analysis (PCA) of the TCR clonality and TRB V gene usages, coloured by patient group.

c) TRBV proportions between ME and AE patient groups of total tumour T cells, activated Tregs Treg cells and CD8 activated EM T cells within the PancrImmune dataset. Orange represents ME patients and grey represents AE patients.

d) Clonality of the blood T cell subpopulations between the ME and AE patient groups via two measures: (top) *intra-subset clonality* (the percentage of cells in clones >2 cells per subset, measuring the clonality within the subset thus reflecting specific cell populations which are actively expanding), and (bottom) *inter-subset clonality* (the percentage of cells of each cell type as members of clones >3 cells across all populations, demonstrating, this indicates cells within each T cell subset that may be members of larger clones than span multiple phenotypes, reflecting T cell plasticity of expanding clones).

e) The *inter-subset clonality* between tumour (blue) and blood (red) T cells.

f) The percentage of TCRs from each T cell subset that match to anti-viral T cell clones, coloured by patient group.

All analyses in panels b-d were performed on intra-tumoural cells in the PancrImmune dataset, and panels e-f were performed on intra-tumoural or blood cells in the PancrImmune dataset * denotes p-values<0.05, and tests were performed by two-sided MANOVA. ME patients have an n=5 and AE patients have an n=7.

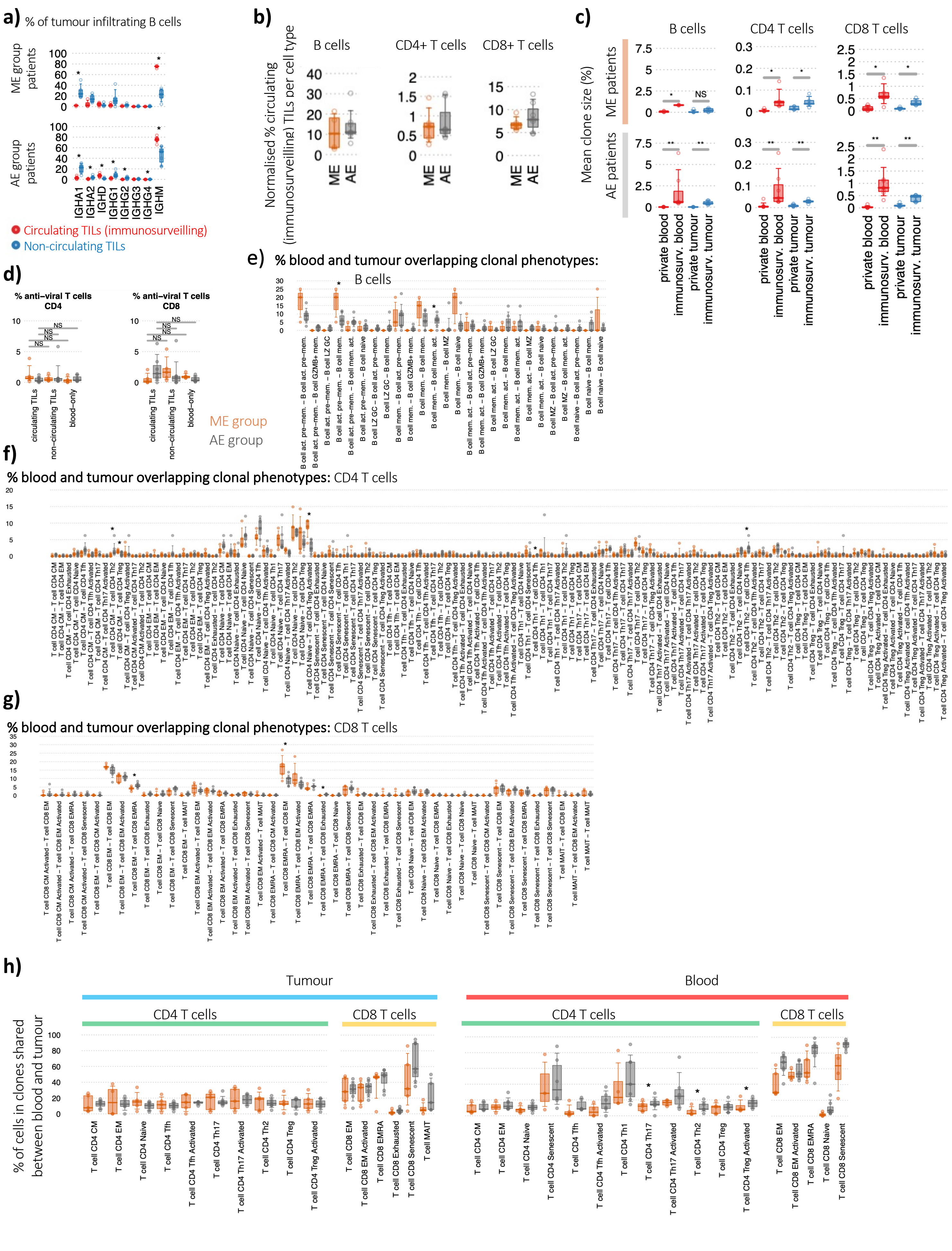

Supplementary Figure 12

Supplementary Figure 12: Immunosurveilling and resident B and T cell clones are phenotypically distinct.
a) The isotype usage percentages of tumour B cells have clonal members in the blood or (blue) no clonal members in the blood between ME patients (top) and AE patients (bottom).
b) The normalised level of re-circulating tumour clones between ME and AE patients B and T cell clones.
c) The mean clone sizes per patient between blood and tumour re-circulating and private clones, plotted between ME and AE patient groups. * denotes p-values<0.05, ** p-values<0.005.
d) The percentage of TCRs that match to anti-viral T cell clones between re-circulating and private clones, coloured by patient group.
The relative proportions of clones overlapping blood and tumour split by phenotype for e) B cells, f) CD4 T cells, and g) CD8 T cells, coloured by patient group
h) The percentage of cells in clones shared between blood and tumour, split by source and coloured by patient group.
All analyses in this figure were performed on the PancrImmune dataset using both the blood and tumour samples. Unless otherwise mentioned, * denotes p-values<0.05 and tests were performed by two-sided MANOVA. ME patients have an n=5 and AE patients have an n=7.

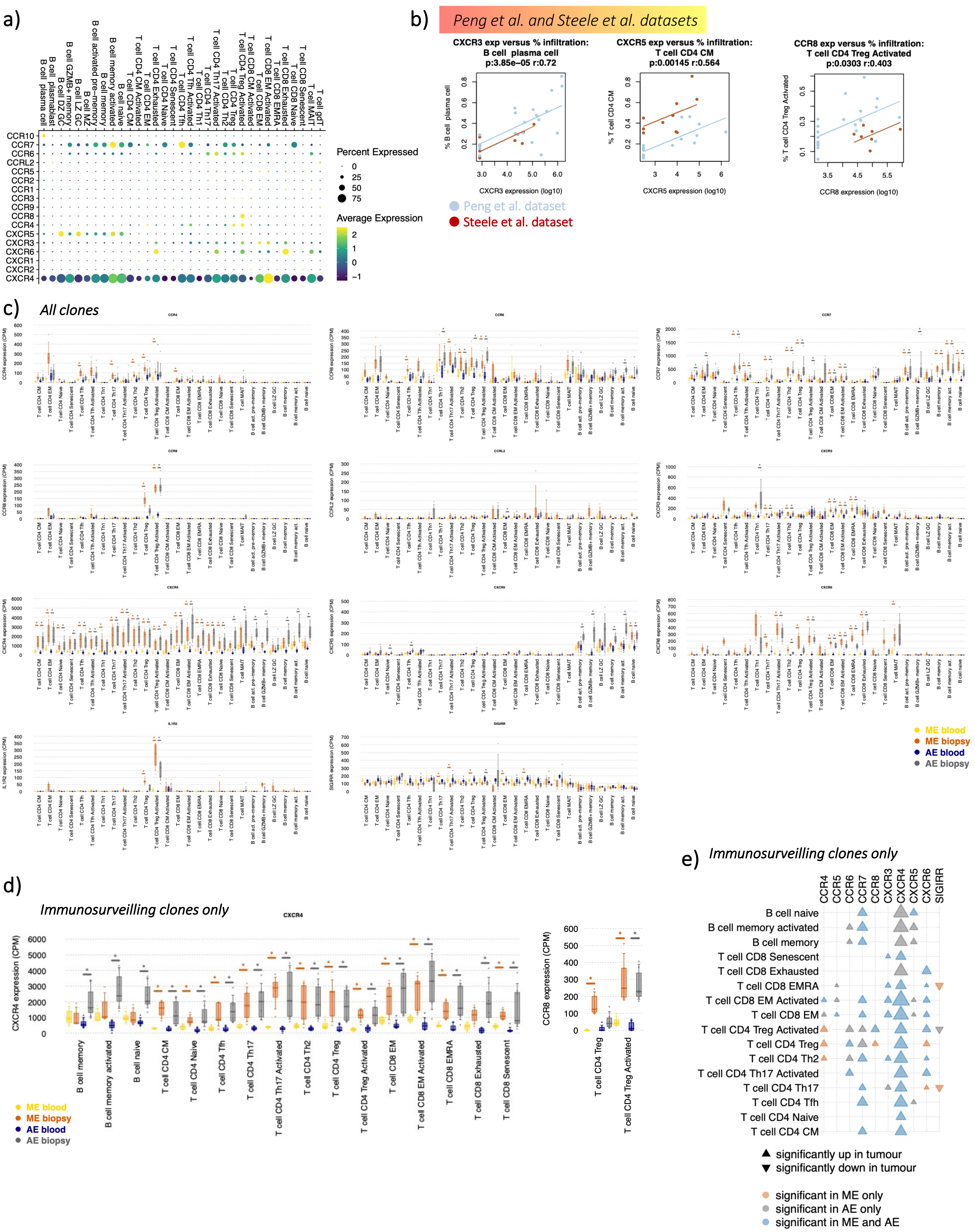

Supplementary Figure 13

Supplementary Figure 13: Distinct chemokine receptor expression between ME and AE groups.
a) Gene expression dot plot of the chemokine receptor gene expression across lymphocytes.
b) Correlations between immune cell proportion within tumour microenvironment and mean chemokine expression of that cell type. P-values and r^2 values were computed using the repeated measures correlation from rmcorr package in R, with the patients from the Peng et al. data in blue and the Steele et al. data in red.
c) The mean gene expression of key lymphocyte chemokine receptors between all blood and tumour biopsy between ME and AE patients for each cell type. Each point represents the mean expression per patient per cell group.
d) The mean gene expression of key CXCR4 and CCR8 between blood and tumour biopsy between ME and AE patients for each cell type for only immunosurveilling clones (clones shared between blood and tumour). Each point represents the mean expression per patient per cell group. * denotes p-values <0.05 as determined by DGE.
e) Heatmap of DGE between blood and tumour biopsy for immunosurveilling clones (clones shared between blood and tumour) between ME and AE patients. For each chemokine receptor and for each cell type, the upwards triangle denotes significant elevation of expression in tumour compared to blood and downwards triangle denotes significant reduction of expression in tumour compared to blood. The triangles are coloured orange, grey and blue if the significance is observed in ME patients only, AE patients only or both, respectively. The sizes of the triangles denote relative mean expression.
All analyses in this figure were performed on the PancrImmune dataset using both the blood and tumour samples. Unless otherwise indicated, * denotes p-values<0.05 and tests were performed by two-sided MANOVA. ME patients have an n=5 and AE patients have an n=7.

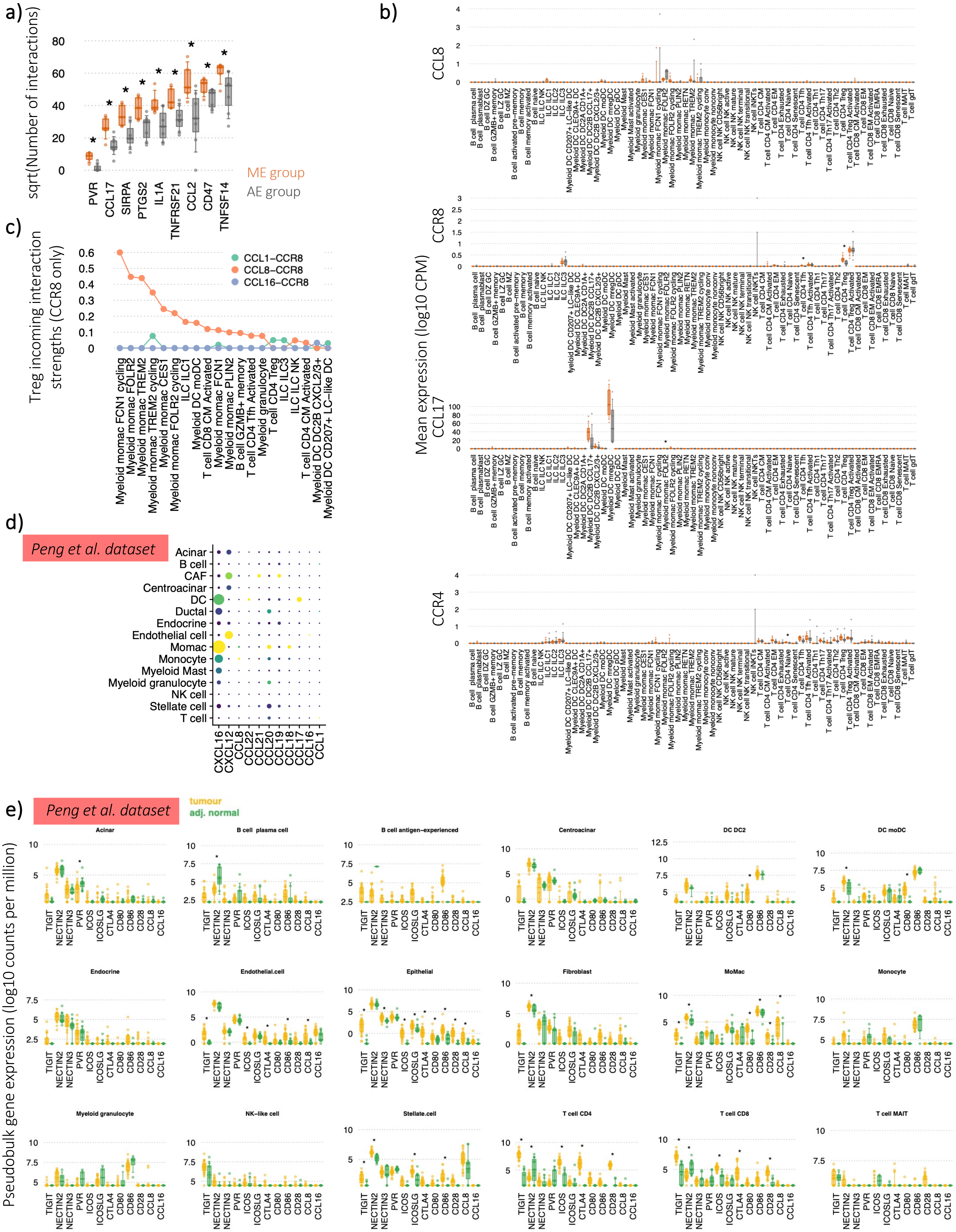

Supplementary Figure 14

Supplementary Figure 14: Distinct regulatory mechanisms between patients with different immune cell infiltration.

a) The number of interactions of the nine significantly enriched immune modulators (p-values<0.05) are known chemokines associated with immunosuppression or apoptosis (p-values<0.05).

b) The mean normalised gene expression per sample of key Treg chemokines and their receptors across cell types within the tumour from the PancrImmune dataset. * denotes p-values<0.05 and tests were performed by two-sided MANOVA.

c) The top 20 ranked interaction strengths between the exclusive Treg receptor CCR8 and its ligands per cell type, coloured by receptor-ligand interaction type.

d) The relative expression of key lymphocyte chemokines across both immune and non-immune cells, using the Peng dataset. The circle colour denotes relative mean expression level (yellow indicates higher levels) and size indicates percentage of cells expressing each gene.

e) Boxplots differential checkpoint gene expression between adjacent normal pancreatic tissue and PDAC in both immune and non-immune cell compartments using the Peng dataset.

All analyses were performed on intra-tumoural cells. ME patients have an n=5 (PancrImmune) and n=11 (Peng), and AE patients have an n=7 (PancrImmune) and n=13 (Peng).
