# Supplementary material for: Distinct immune cell infiltration patterns in pancreatic ductal adenocarcinoma (PDAC) exhibit divergent immune cell selection and immunosuppressive mechanisms
Source: Nat Commun. 2025 Feb 6;16:1397. doi: 10.1038/s41467-024-55424-2 (PMC11802853; doi:10.1038/s41467-024-55424-2)
Supplement: Supplementary file 4 — Supplementary Data 1 [file 41467_2024_55424_MOESM4_ESM.pdf]

Title: Distinct immune cell infiltration patterns in pancreatic ductal adenocarcinoma (PDAC) exhibit divergent immune cell selection and immunosuppressive mechanisms

Shivan Sivakumar[*1,2,3], Ashwin Jainarayanan[*2,4], Edward Arbe-Barnes[*5], Piyush Kumar Sharma[+1], Maire Ni Leathlobhair[+6,7], Sakina Amin[+8], David J Reiss[+9], Lara Heij[10,11], Samarth Hegde[12], Assaf Magen[12], Felicia Tucci[8,13,14], Bo Sun[15], Shihong Wu[8,13], Nithishwer Mouroug Anand[8], Hubert Slawinski[14], Santiago Revale[14], Isar Nassiri[14], Jonathon Webber[2], Gerard D. Hoeltzel[8], Adam Frampton[16,17,18,19], Georg Wiltberger[20], Ulf Neumann[21], Philip Charlton[1], Laura Spiers[1], Tim Elliott[22], Maria Wang[23], Suzana Couto[24], Thomas Lila[23], Pallavur V. Sivakumar[9], Alexander V. Ratushny[9], Mark Middleton[1], Dimitra Peppa[25,26], Benjamin Fairfax[1], Miriam Merad[12], Michael L. Dustin[2,27§], Enas Abu-Shah[2,28§], Rachael Bashford-Rogers[8,13,14§]

Supplemental Item 1.

Figure 1. Gene signatures for the myeloid cell annotations
a) Tumour myeloid UMAP coloured by (i) patient, and (ii) broad myeloid cell type
Gene expression signatures of (b) broad myeloid cell types, (c) DC, (d) monocyte-derived macrophages (momac), (e) mast cell, (f) monocyte, and g) ILC subpopulations.
The suffix "b" indicates blood-derived populations.

Figure 2. Gene signatures for the T/NK cell annotations
a) UMAP coloured by sample source.
b) (i) CITE-seq and (ii) gene expression signatures of NK versus T cells.
c) (i) CITE-seq and (ii) gene expression signatures of CD4 versus CD8 T cells.
d) CITE-seq signatures of (i) CD4 T cell, (ii) CD8 T cell and (iii) NK cell populations. CITE-seq values are scaled by maximum value per cell type group.
e) Gene expression signatures of (i) CD4 T cell, (ii) CD8 T cell and (iii) NK cell populations.

Figure 3. Gene and VDJ signatures for the B cell annotations.
a) UMAP plots of B cells coloured by (i) source, (ii) somatic hypermutation level, (iii) isotype and (iv) VDJ expression level.
b) Correlation of IGH and IGK/L UMI counts per cell, coloured by PDAC sample type.
c) The per cell subpopulation (i) somatic hypermutation levels and ii) VDJ expression level.
d) The isotype usage percentages across cell types within each cell population.
e) Gene expression profiles of B cell subpopulations of the top differentially expressed genes.

Figure 4. Clone size distributions across cell types for (left) B cell and (right) T cell populations, considering only intra-tumoural immune cells.

Figure 5
a) Correlation of cell types between technical repeats of biopsy samples for the broad immune cell subsets, B cell subsets, T cell subsets and myeloid cell subsets.
b) Boxplots of differences between biopsy and blood immune cell proportions. Each dot represents a patient sample.

Figure 6. a) Schematic of SVMCellTransfer alongside the advantages of this method over established methods (left).
b) Comparison of reference annotation of T and NK cells using SVMCellTransfer (using T and NK cell PancrImmune reference), and Azimuth annotation (using either pancreas or PBMC references). High-confidence T and NK cells were subsampled from the Peng and Steele datasets (confirmed by manual checking of key T and NK genes), and applied both the SVMCellTransfer (using T and NK cell PancrImmune reference), and Azimuth annotation (using either pancreas or PBMC references). UMAP plots show the distribution of predicted cell types by each method.

Figure 7. UMAP distributions of the integrated PDAC150K, Peng, et al. and Steele, et al. datasets.

Figure 8. Gene expression signatures of (ai) T and NK cell types, (ii) DC, (d) myeloid cell types, (iii) B cell subtypes, (bi) non-immune cell types, and (ii) CAF cell types of the integrated PDAC150K, Peng, et al. and Steele, et al. datasets.

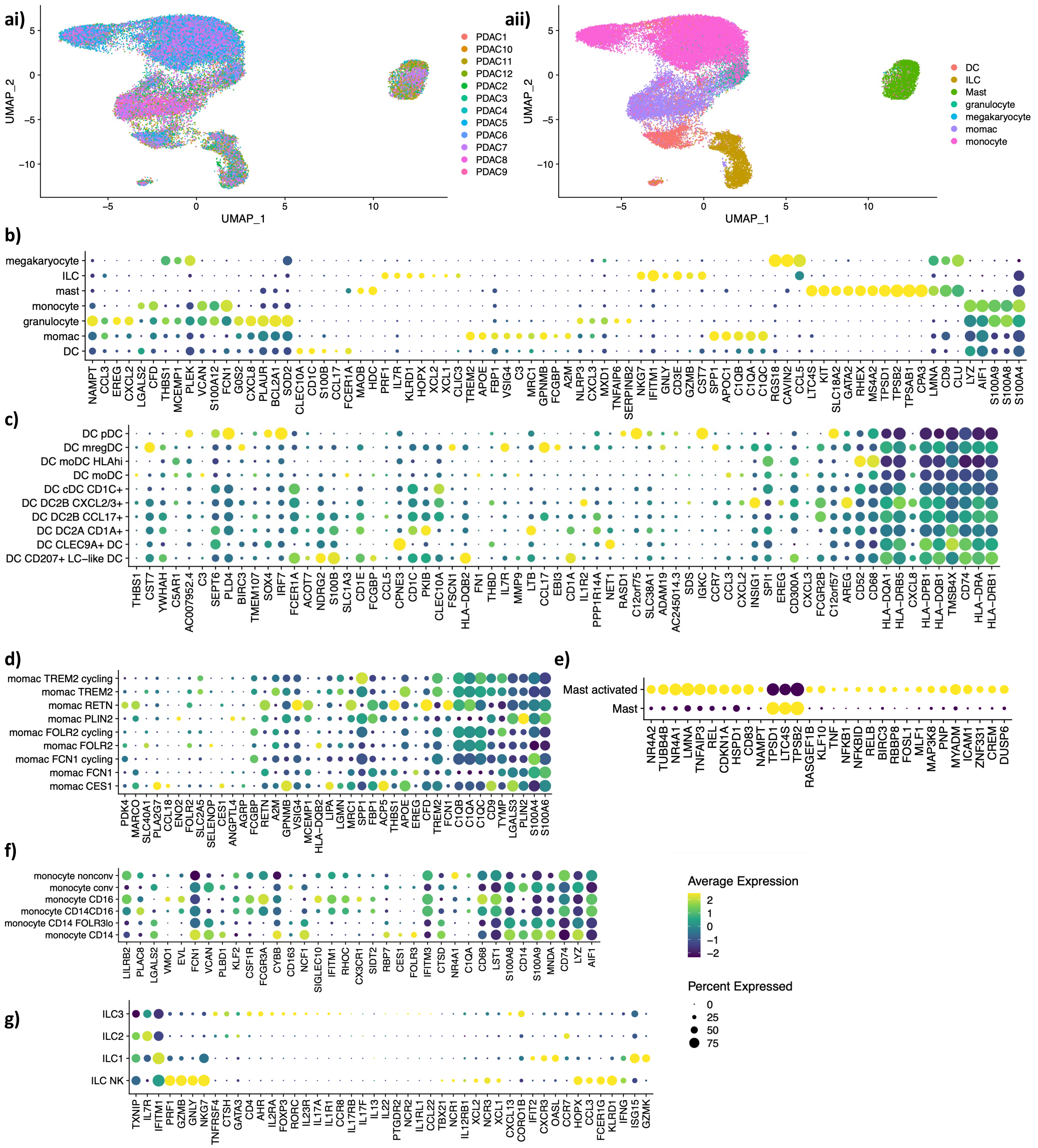

Supplemental Data Figure 1.

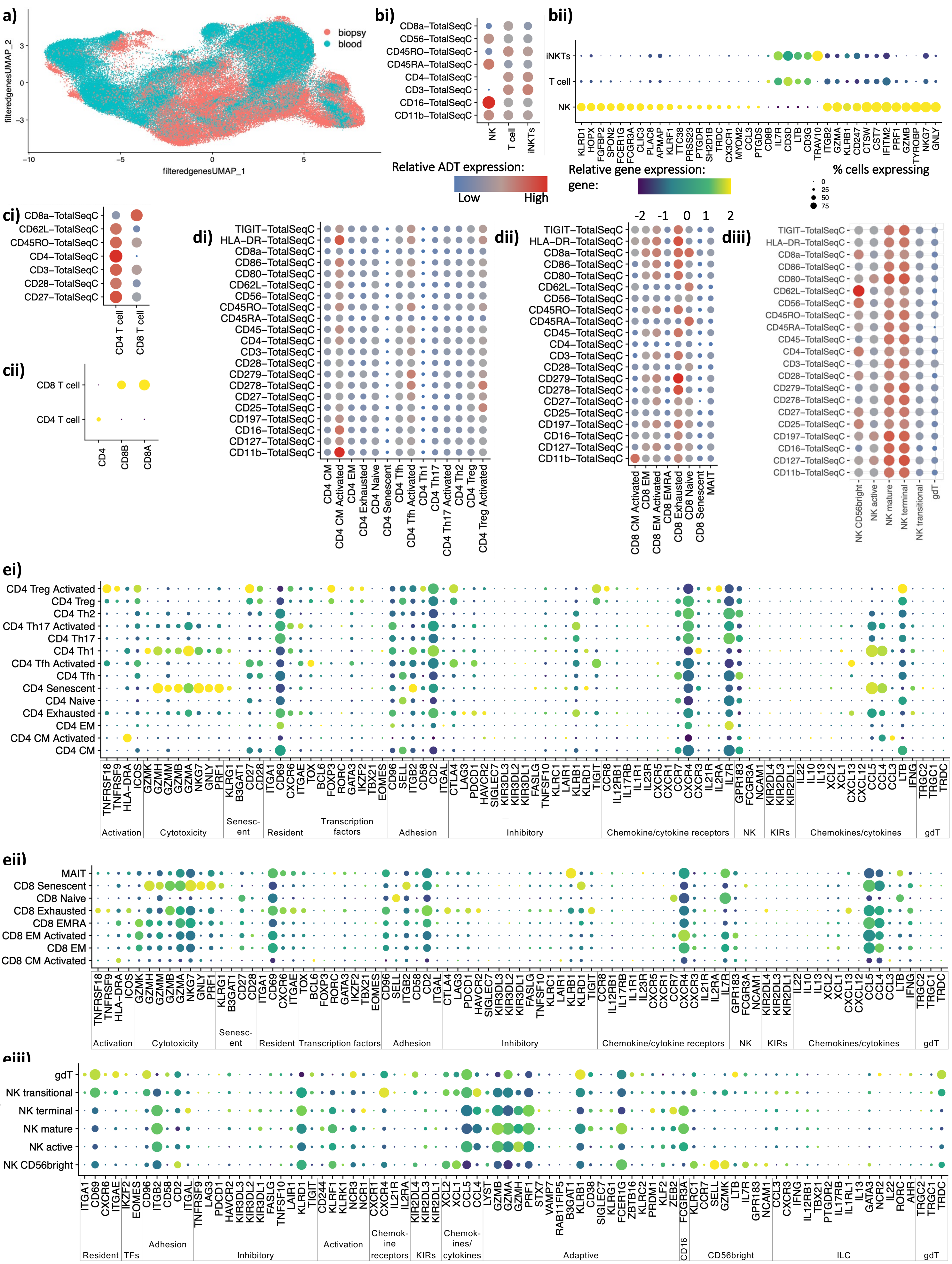

Supplemental Data Figure 2.

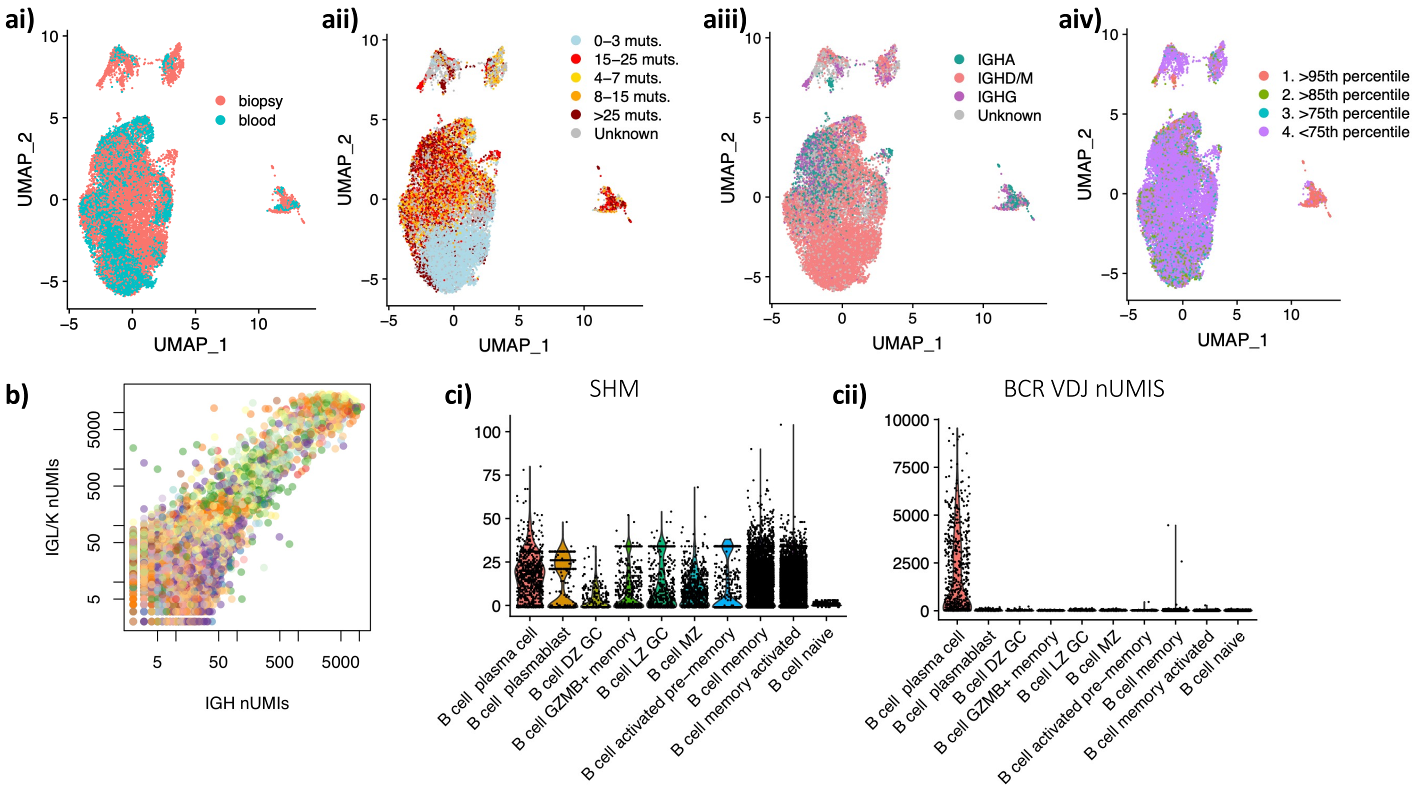

d) % B cells per isotype per cell type

	IGHA1	IGHA2	IGHD/M	IGHG1	IGHG2	IGHG3	IGHG4
B cell plasma cell	27.09	15.98	20.09	25.57	8.07	2.74	0.46
B cell plasmablast	2.21	23.53	10.29	62.13	1.10	0.74	0.00
B cell DZ GC	2.31	0.00	42.31	43.08	3.85	7.69	0.77
B cell GZMB+ memory	10.54	3.43	78.19	6.37	0.00	1.47	0.00
B cell LZ GC	2.42	0.66	55.16	31.87	5.71	3.08	1.10
B cell MZ	9.58	0.63	76.45	8.01	0.63	4.71	0.00
B cell activated pre-memory	6.96	0.56	88.58	3.06	0.28	0.56	0.00
B cell memory	15.76	4.25	67.18	7.90	3.41	1.10	0.40
B cell memory activated	24.66	4.83	61.24	6.81	1.78	0.63	0.06
B cell naive	0.00	0.00	100.00	0.00	0.00	0.00	0.00

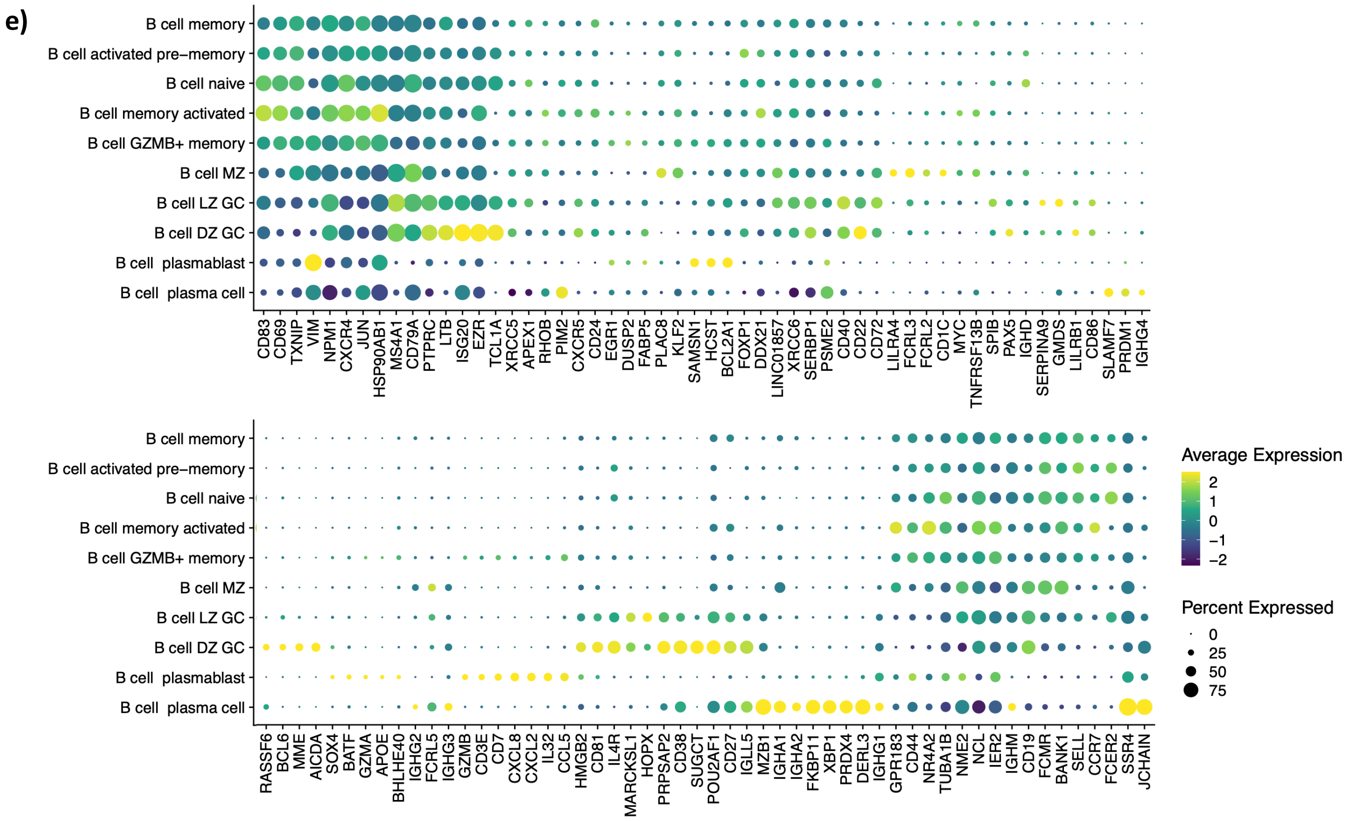

Supplemental Data Figure 3.

Clone size distributions across cell types (intra-tumoural samples)

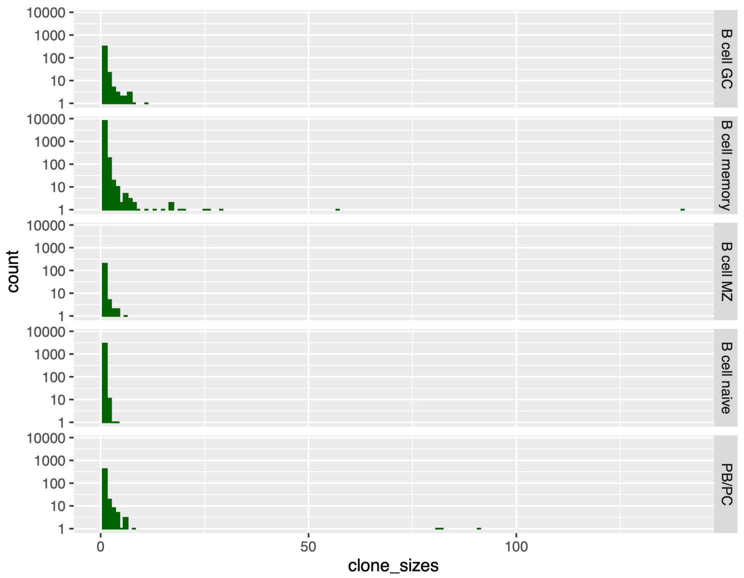

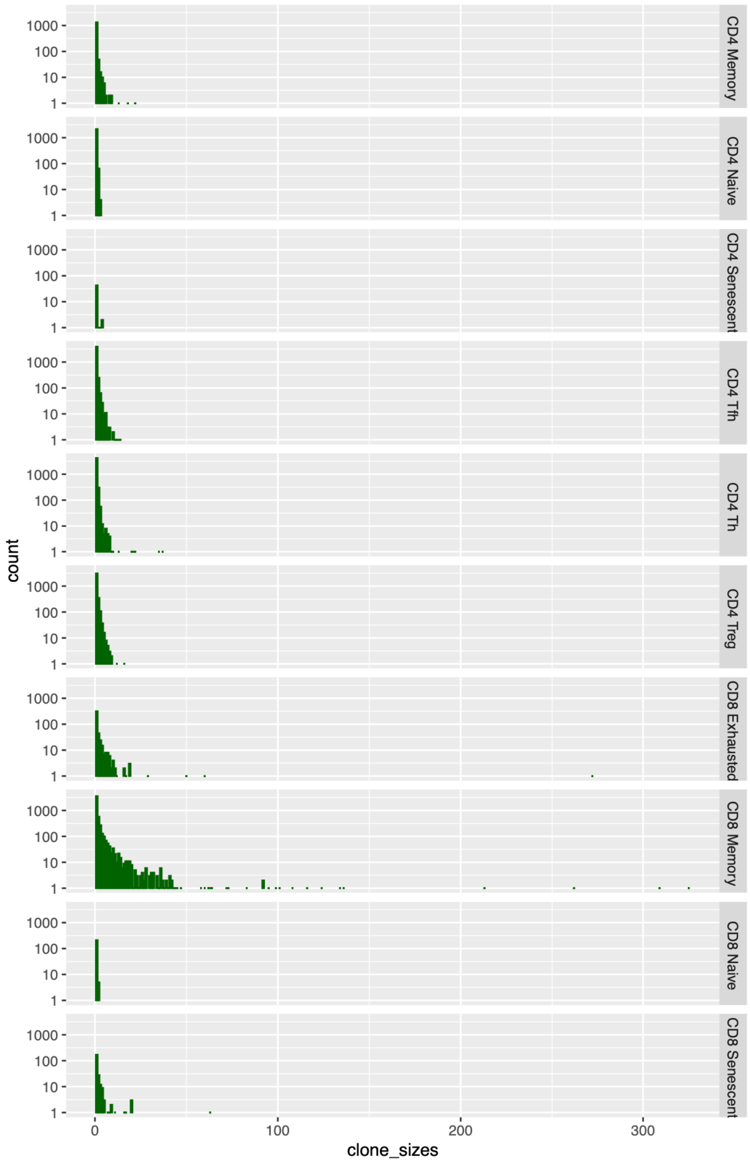

Supplemental Data Figure 4.

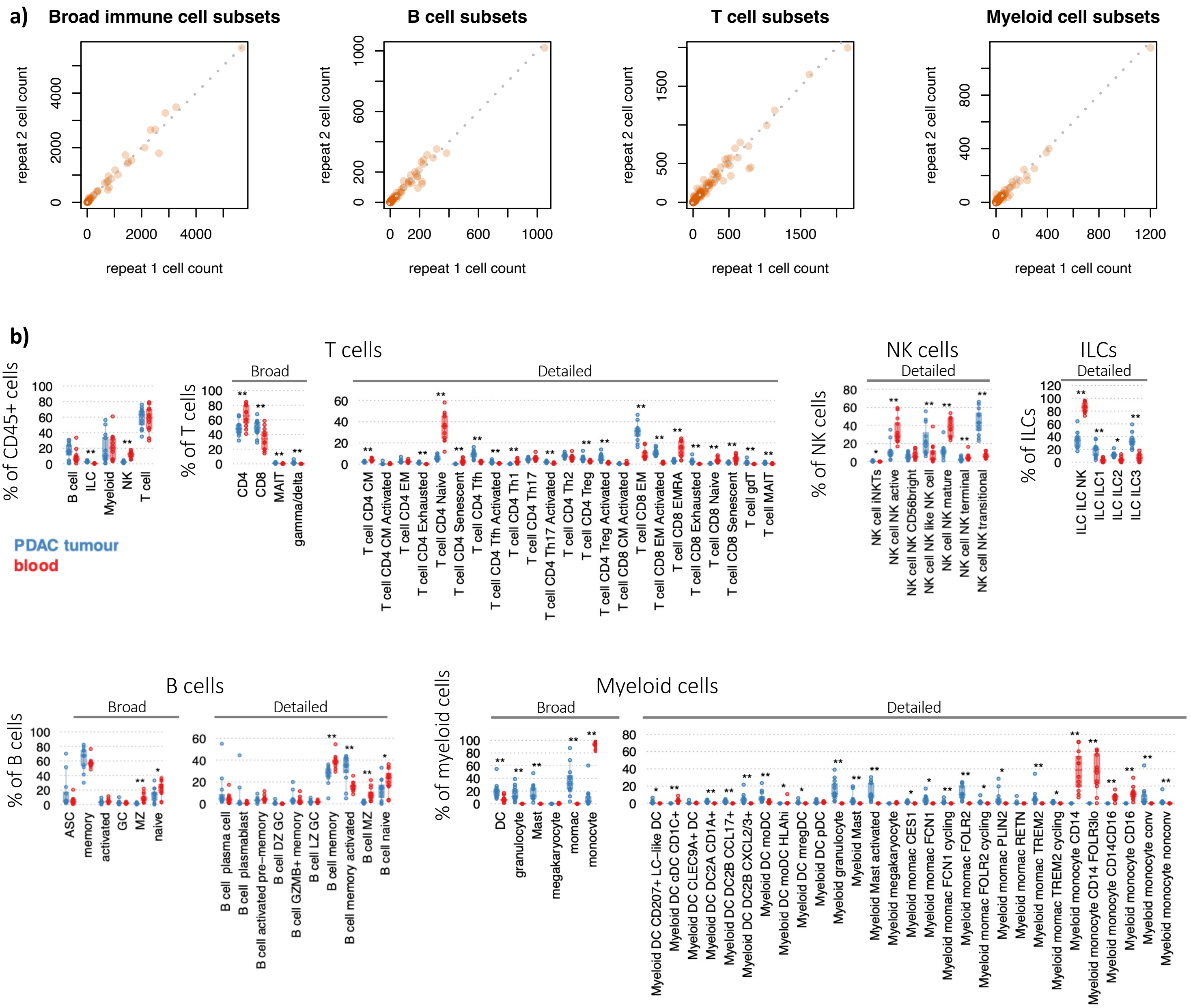

Supplemental Data Figure 5.

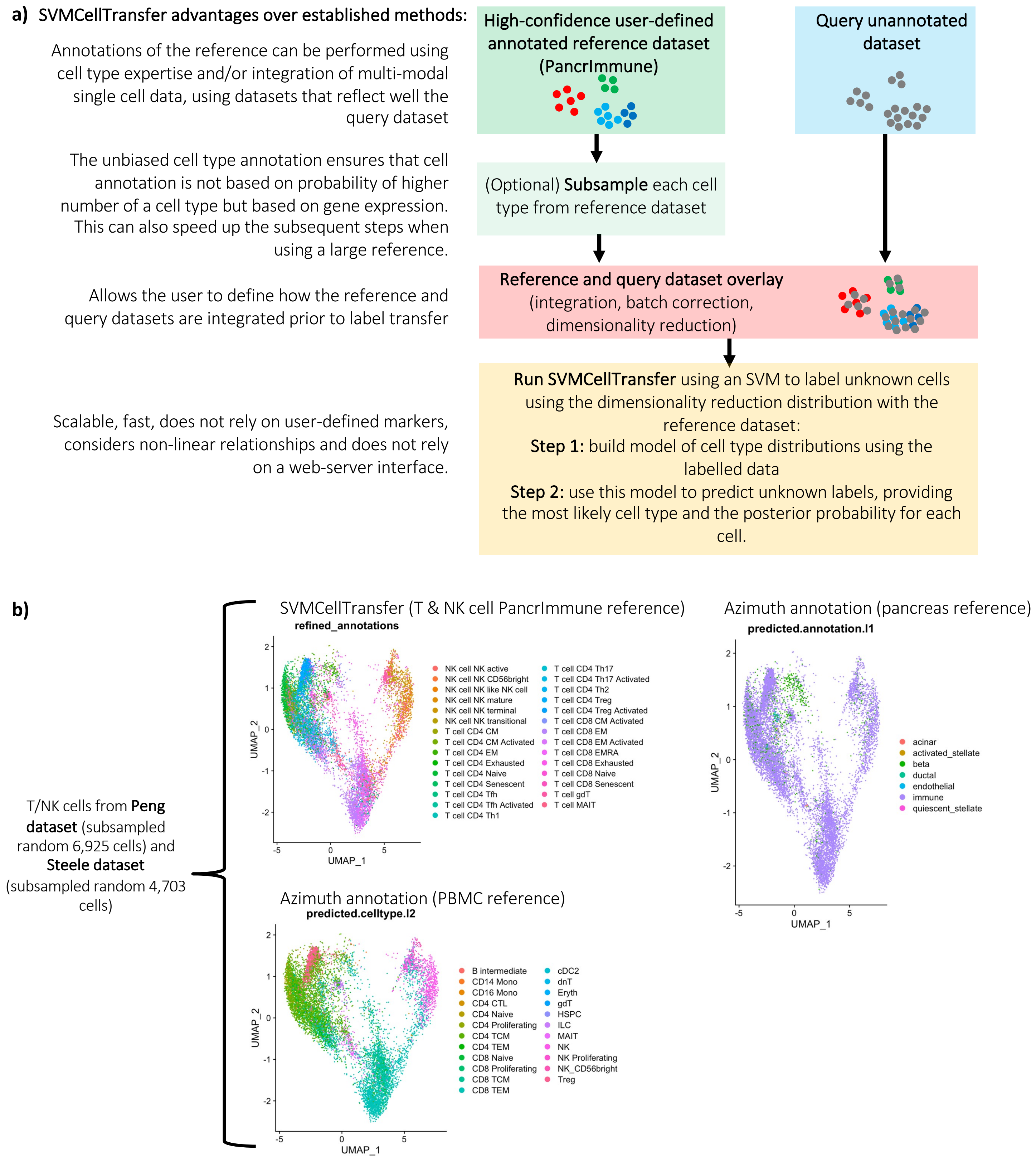

Supplemental Data Figure 6.

Integrated PDAC150K, Peng, *et al.* and Steele, *et al.* datasets

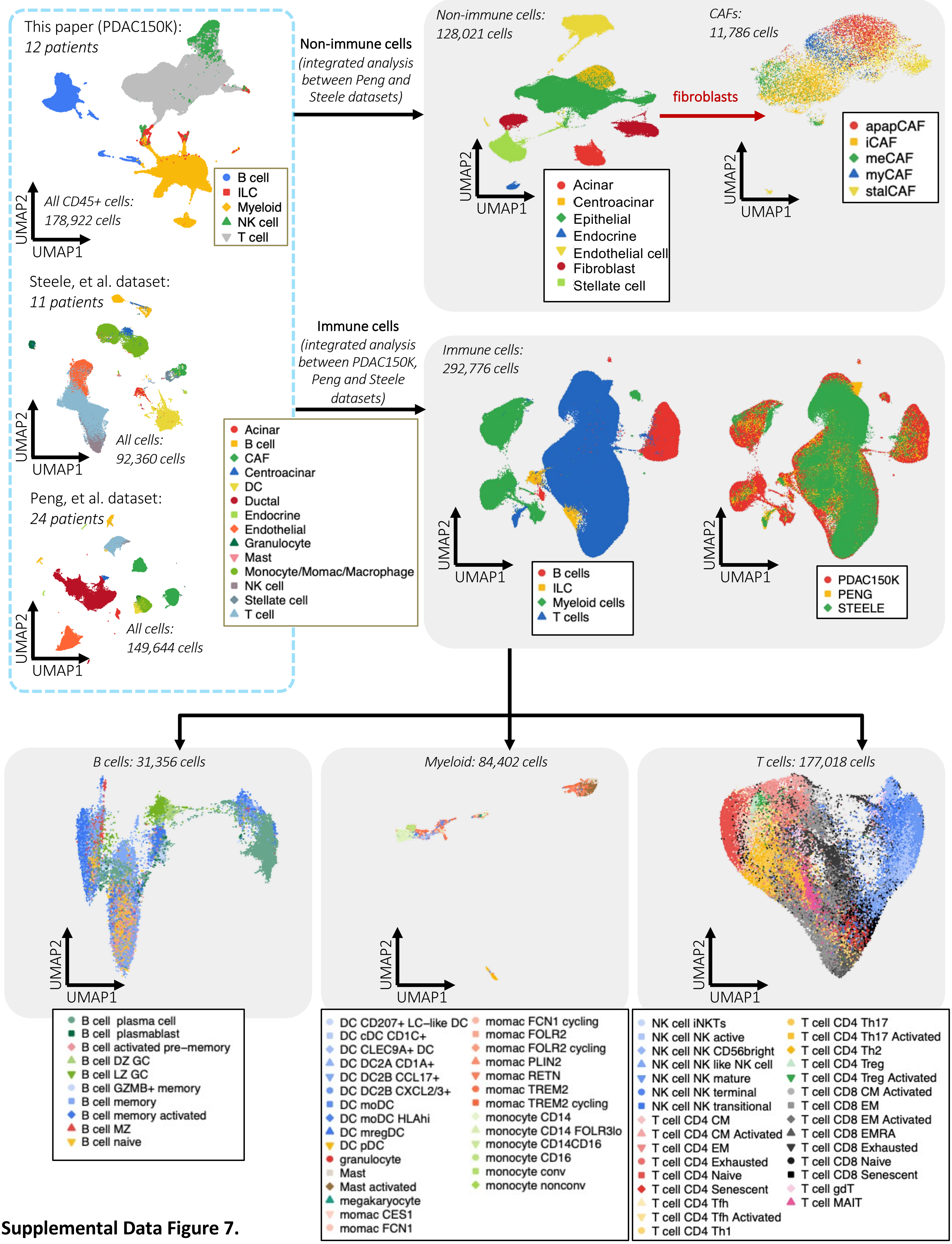

Supplemental Data Figure 7.

ai) Immune cells *(integrated analysis between PDAC150K, Peng and Steele datasets)*

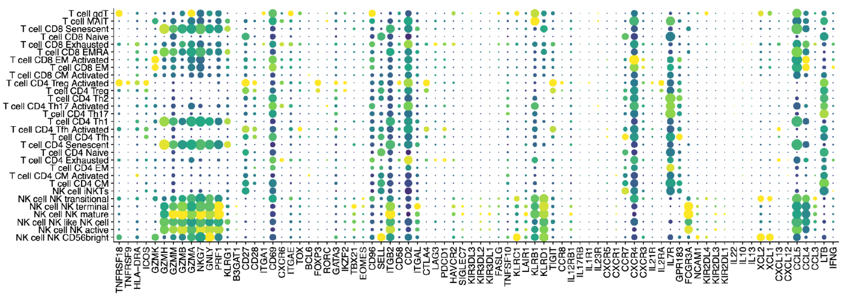

ii)

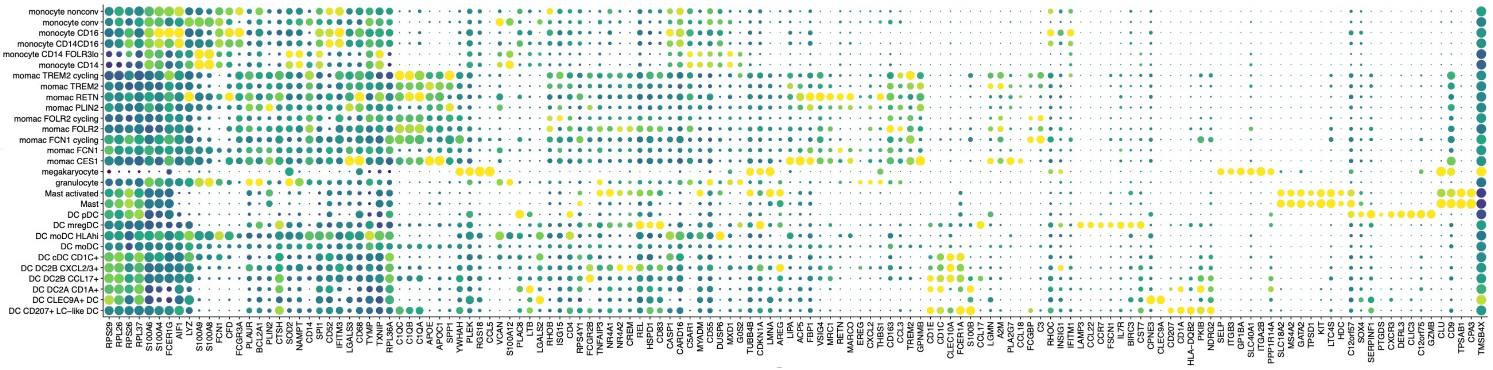

iii)

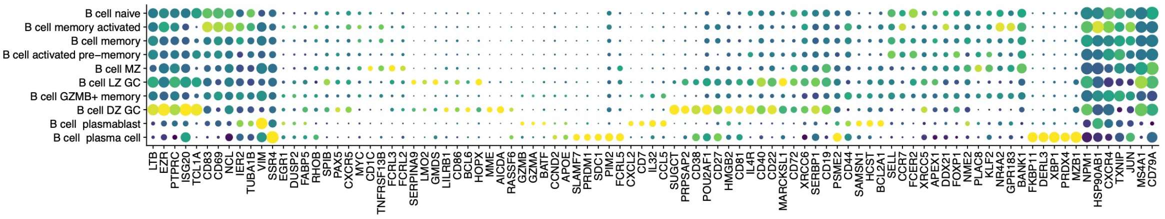

bi) Non-immune cells *(integrated analysis between Peng and Steele datasets)*

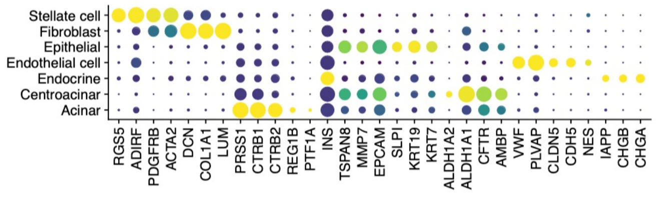

ii)

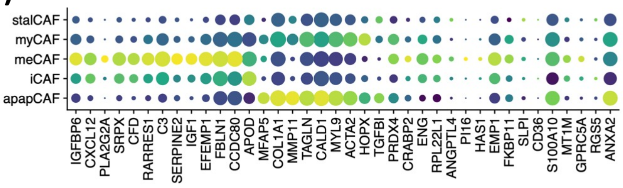

Supplemental Data Figure 8.
