# Supplementary material for: Distinct immune cell infiltration patterns in pancreatic ductal adenocarcinoma (PDAC) exhibit divergent immune cell selection and immunosuppressive mechanisms
Source: Nat Commun. 2025 Feb 6;16:1397. doi: 10.1038/s41467-024-55424-2 (PMC11802853; doi:10.1038/s41467-024-55424-2)
Supplement: Supplementary file 15 — Reporting Summary [file 41467_2024_55424_MOESM15_ESM.pdf]

Corresponding author(s): Shivan Sivakumar, Enas Abu-Shah, Rachael Bashford-Rogers

Last updated by author(s): 27/03/2024

Reporting Summary

Nature Portfolio wishes to improve the reproducibility of the work that we publish. This form provides structure for consistency and transparency in reporting. For further information on Nature Portfolio policies, see our Editorial Policies and the Editorial Policy Checklist.

Statistics

For all statistical analyses, confirm that the following items are present in the figure legend, table legend, main text, or Methods section.

n/a	Confirmed	
☐	☒	The exact sample size (*n*) for each experimental group/condition, given as a discrete number and unit of measurement
☐	☒	A statement on whether measurements were taken from distinct samples or whether the same sample was measured repeatedly
☐	☒	The statistical test(s) used AND whether they are one- or two-sided<br>*Only common tests should be described solely by name; describe more complex techniques in the Methods section.*
☐	☒	A description of all covariates tested
☐	☒	A description of any assumptions or corrections, such as tests of normality and adjustment for multiple comparisons
☐	☒	A full description of the statistical parameters including central tendency (e.g. means) or other basic estimates (e.g. regression coefficient) AND variation (e.g. standard deviation) or associated estimates of uncertainty (e.g. confidence intervals)
☐	☒	For null hypothesis testing, the test statistic (e.g. *F*, *t*, *r*) with confidence intervals, effect sizes, degrees of freedom and *P* value noted<br>*Give P values as exact values whenever suitable.*
☒	☐	For Bayesian analysis, information on the choice of priors and Markov chain Monte Carlo settings
☒	☐	For hierarchical and complex designs, identification of the appropriate level for tests and full reporting of outcomes
☐	☒	Estimates of effect sizes (e.g. Cohen's *d*, Pearson's *r*), indicating how they were calculated

Our web collection on statistics for biologists contains articles on many of the points above.

Software and code

Policy information about availability of computer code

Data collection	Clinical data was collected in Microsoft Excel.
Data analysis	All code is available via https://github.com/rbr1/scIsoTyper/, https://github.com/rbr1/PancrImmune_PDAC_paper, and https://github.com/sakinaamin/BayesPrism.<br><br>List of software used:<br>• IMGT/HighV-QUEST: version 1.8.5<br><br>R version 4.3.0 and associated packages:<br>• ape: version 5.6<br>• caret: version 6.0-90<br>• corrplot: version 0.92<br>• edgeR: version 3.32.1<br>• fgsea: version 1.16.0<br>• igraph: version 1.6.0<br>• phangorn: version 2.7.1<br>• vegan: version 2.5-7<br>• RColorBrewer: version 1.1-3<br>• BayesPrism: version 2.0

- Seurat: version 5.0.1
- survival: version 3.5-7
- psych: version 2.4.1
- harmony: version 1.2.0
- ggplot2: version 3.4.4
- future: version 1.33.1
- pryr: version 0.1.6

For manuscripts utilizing custom algorithms or software that are central to the research but not yet described in published literature, software must be made available to editors and reviewers. We strongly encourage code deposition in a community repository (e.g. GitHub). See the Nature Portfolio guidelines for submitting code & software for further information.

Data

Policy information about availability of data

All manuscripts must include a data availability statement. This statement should provide the following information, where applicable:

- Accession codes, unique identifiers, or web links for publicly available datasets
- A description of any restrictions on data availability
- For clinical datasets or third party data, please ensure that the statement adheres to our policy

Data is available in study SCP2405 in the Single Cell Portal (https://singlecell.broadinstitute.org) and study 7425252850300752366-242ac117-0001-012 in the VDJserver (https://vdjserver.org/).

Research involving human participants, their data, or biological material

Policy information about studies with human participants or human data. See also policy information about sex, gender (identity/presentation), and sexual orientation and race, ethnicity and racism.

Reporting on sex and gender	This is provided in Table S1.
Reporting on race, ethnicity, or other socially relevant groupings	This data was not available for the patients in this study. However, 3 cohorts were used from a UK-based study (PancrImmune), Chinese-based study (Peng), and a US-based study (Steele), thus likely to be representative of wider ethnic backgrounds.
Population characteristics	PDAC patients undergoing surgical resection or biopsy.
Recruitment	Multi-centre
Ethics oversight	The study was in strict compliance with all institutional ethical regulations.

Note that full information on the approval of the study protocol must also be provided in the manuscript.

Field-specific reporting

Please select the one below that is the best fit for your research. If you are not sure, read the appropriate sections before making your selection.

☒ Life sciences ☐ Behavioural & social sciences ☐ Ecological, evolutionary & environmental sciences

For a reference copy of the document with all sections, see nature.com/documents/nr-reporting-summary-flat.pdf

Life sciences study design

All studies must disclose on these points even when the disclosure is negative.

Sample size	ME patients have an n=5 (PancrImmune), n=11 (Peng) and n=7 (Steele), and AE patients have an n=7 (PancrImmune), n=13 (Peng) and n=4 (Steele).
Data exclusions	Not applicable. Sequencing was performed in all cases where tissue was acquired.
Replication	For ten of the tumour samples, two replicate 10X scRNA-seq, VDJ-seq and CITE-seq runs were performed. High levels of correlation between cell type proportions and VDJ clonality were observed in these replicates.
Randomization	Randomisation not applicable - all cases were treated with standard of care therapy regimens.
Blinding	Blinding not applicable - no group allocations.

Reporting for specific materials, systems and methods

We require information from authors about some types of materials, experimental systems and methods used in many studies. Here, indicate whether each material, system or method listed is relevant to your study. If you are not sure if a list item applies to your research, read the appropriate section before selecting a response.

Materials & experimental systems

n/a	Involved in the study
☐	☒ Antibodies
☒	☐ Eukaryotic cell lines
☒	☐ Palaeontology and archaeology
☒	☐ Animals and other organisms
☐	☒ Clinical data
☒	☐ Dual use research of concern
☒	☐ Plants

Methods

n/a	Involved in the study
☒	☐ ChIP-seq
☒	☐ Flow cytometry
☒	☐ MRI-based neuroimaging

Antibodies

Antibodies used	anti-CD45 antibody diluted at 1:200 (Clone 2D1 PECy7, 368532) Biolegend
Validation	*Describe the validation of each primary antibody for the species and application, noting any validation statements on the manufacturer's website, relevant citations, antibody profiles in online databases, or data provided in the manuscript.*

Clinical data

Policy information about clinical studies

All manuscripts should comply with the ICMJEguidelines for publication of clinical research and a completedCONSORT checklist must be included with all submissions.

Clinical trial registration	The clinical trial discussed is fully published: Tempero, M. A. et al. Adjuvant nab-Paclitaxel + Gemcitabine in Resected Pancreatic Ductal Adenocarcinoma: Results From a Randomized, Open-Label, Phase III Trial. J Clin Oncol 41, 2007-2019 (2023). https://doi.org:10.1200/JCO.22.01134
Study protocol	For single cell data, the ethics used were Oxford Radcliffe biobank (09/H0606/5+5, project: 19/A177) and Aachen medical centre under RWTH Aachen biobank project: EK360/19.
Data collection	*Describe the settings and locales of data collection, noting the time periods of recruitment and data collection.*
Outcomes	*Describe how you pre-defined primary and secondary outcome measures and how you assessed these measures.*

Plants

Seed stocks	*Report on the source of all seed stocks or other plant material used. If applicable, state the seed stock centre and catalogue number. If plant specimens were collected from the field, describe the collection location, date and sampling procedures.*
Novel plant genotypes	*Describe the methods by which all novel plant genotypes were produced. This includes those generated by transgenic approaches, gene editing, chemical/radiation-based mutagenesis and hybridization. For transgenic lines, describe the transformation method, the number of independent lines analyzed and the generation upon which experiments were performed. For gene-edited lines, describe the editor used, the endogenous sequence targeted for editing, the targeting guide RNA sequence (if applicable) and how the editor was applied.*
Authentication	*Describe any authentication procedures for each seed stock used or novel genotype generated. Describe any experiments used to assess the effect of a mutation and, where applicable, how potential secondary effects (e.g. second site T-DNA insertions, mosiacism, off-target gene editing) were examined.*
